# Supplementary material for: Speciation with gene flow via cycles of isolation and migration: insights from multiple mangrove taxa
Source: Natl Sci Rev. 2018 Jul 24;6(2):275–88. doi: 10.1093/nsr/nwy078 (PMC6599600; doi:10.1093/nsr/nwy078)
Supplement: Supplemental Files [file nwy078_supplemental_files.zip › nwy078_supplement_0404.pdf]

Supplementary Information for

Speciation with gene flow via cycles of isolation and migration: Insights from multiple mangrove taxa

This file includes:

Supplementary Note
Tables S1-S6
Figures S1-S18
References 93-117

Supplementary Note

Replies to objections to the MIM model

As the study has been widely circulated prior to the submission to National Science Review, it has received many comments and criticisms. During the reviewing process, there are additional comments. Since these comments likely represent common opinions and the main text cannot address them in detail, we respond to the criticisms here in the supplement. These criticisms fall into two major categories:

1. Allopatry, or strict geographical isolation, does not reflect the current thinking. Some reviewers even argue that it never did.

Reply **-** Although there are always skeptics about the view of allopatry, there is no denial that it has been the dominant perspective. All textbooks on evolution and specialized books on speciation attest to the dominance. The detailed reply #1 below addresses this issue. It is indeed true that the current thinking has veered somewhat toward the acceptance of "speciation with gene flow". The detailed reply #2 points out that the proposal of "speciation with gene flow" has failed to reject the classical view of allopatry. So, we are back to square one of testing allopatry against observations.

2. The MIM view has not been rigorously modeled and may not be new.

Reply **-** The detail replies #3, #4 and #5 respond to the criticism. Like many view points in biology, it is the strength (as well as the type) of evidence supporting a particular view that is novel, rather than the view point itself.

The MIM (for mixing-isolation-mixing) mechanism is a specific type of "speciation with gene flow". Despite the many genomic analyses, we have suggested that few have convincingly rejected the model of strict allopatry (i.e., allopatry with no gene flow; Ref.(16)).

To resolve the issue of strict allopatry vs. speciation with gene flow, genic/genomic data are not enough. Other types of data, in particular, the historical records of a well-delineated geographical barrier, are necessary. Furthermore, the genomic data have to come from comparisons that are "older as well as younger" than the speciation event. The combined data are what we present here.

We analyzed mangroves, which have an advantage for the study of speciation: Because mangroves live on the coasts, their geographical distributions are essentially lines, not planes, making the delineation of the barrier much easier. We conclude that speciation in these taxa has proceeded via multiple phases of isolation and migration. Gene flow occurs but phases of strict isolation are also part of the process.

The MIM may be a common, as well as efficient, speciation mechanism because i) many more geographical features, other than something permanent like the Isthmus of Panama, can contribute to speciation; ii) the impermanent closure of the barrier would eventually facilitate species dispersal, thus continually generating new species; iii) postmating isolation in fact requires periods without gene flow to evolve.

Detailed replies to the comments and criticisms, which are given in italics.

1) Point #1 is about the possibility of "speciation with gene flow".

I do not see the conundrum of allopatry as being too inefficient to account for much speciation and indeed, the authors are arguing for a form of allopatry in which barriers are not permanent but repeatedly form and dissipate, as being very efficient for speciation.

Reply #1:

This reviewer is using the concept of allopatry loosely. Allopatry means geographical isolation with no possibility of gene flow. If one allows gene flow (which is against the biological species concept), then the proponents have to define how much and what type of gene flow is permitted. We shall come back to this most central issue below. The reviewer then cited relevant publications in Point #2.

2) Point #2 is about published evidence that speciation has occurred under gene flow.

Others have made the point that geographic barriers are not necessarily permanent and arise a fall through time, resulting in a different evolutionary dynamic than strict allopatric speciation.

Remington long argued for suture zones associated with movement (or not) through mountain passes and Godfrey Hewitt for rapid range expansion of northern species living at the southern edge of Pleistocene glaciers.

As the authors themselves note ... Lake Victoria Cichlids, ... in other systems like Darwin's finches and fly catchers and Rhagoletis etc ... Thus, no one has said that gene flow barriers need be permanent and not fluctuation through time for allopatric speciation to occur.

…Consequently, the current manuscript offers a nice example where this may be the case, but does not offer a novel view of allopatric speciation that solves the geographic problem, as claimed.

Reply #2:

The evidence cited by this reviewer purports to reject the strict allopatric model. These are conclusions based mainly on geographical data. In Coyne and Orr's book as well as in Trevor Price's book, a much larger compilation of such studies fail to convince the authors that the parapatric mode of "speciation with gene flow" is the best explanation.

Conceptually, accepting "speciation with gene flow" is not a small deal for neo-Darwinism as it challenges the fundamental concept of species. Despite the repeated claims of rejecting strict allopatry, the field has provided little convincing evidence that speciation with gene flow has happened with any regularity. This reviewer seems to accept the claim without regard of the extensive literature examined by books dedicated to the study of speciation.

In the recent past, "speciation with gene flow" has been the conclusion of many studies that reported the existence of "genomic islands" (as postulated in the "genic view of speciation"). We published a paper recently (16) with the title "Can the genomic data alone tell us anything about speciation with gene flow?" The answer is "No, they cannot".

A rigorous rejection needs to combine genomic sequences (data of a small fraction of the genome are often insufficient), geographical data, the geological record (of the geographical barrier) and perhaps other ecological observations to accomplish the task. That is what this MIM study does.

3) Point #3 is about the MIM model in relation to the conventional classification of modes of speciation

In this respect, the mixing-isolation-mixing (MIM) model, as described by the authors, requires periods of geographic isolation for speciation to occur. It is not strictly a divergence with gene flow or parapatric model.

Reply #3:

The MIM model is in fact a specific type of parapatric speciation, or speciation with gene flow. The difference is that gene flow happens in episodes, rather than continuously through the whole process. The importance of this mode of gene flow is debated in Point 4.

4) Point #4 is about the reviewer's interpretation of the efficiency of MIM in generating new species.

It is further argued that the MIM model will generate more species than the conventional allopatric model. The idea is that when barriers cyclically form and disappear, taxa can migrate between formerly isolated areas and diverge repeatedly, rather than potentially just once under a "strict" allopatry scenario…

Put another way, the question is whether a situation in which brief periods of isolation that repeatedly occur ... is a more efficient means in generating new taxa than a scenario of permanent isolation … The authors contend the former. However, the answer may be no, ...

Reply #4:

The reviewer mis-interpreted the MIM model. Clearly, we all agree that gene flow would more likely retard divergence than speed it up. What we suggest is that gene flow during speciation may not retard divergence very much as long as selection is driving the divergence (see Fig. 4e, red vs. black lines). This is the original argument of the "genic view of speciation" (6), which applies equally well to the MIM model.

By having a small disadvantage, MIM has many more advantages in generating new species, which are given in response to Point #5 below.

5) Point #5 is an extension of the criticisms of Point #4 above.

Thus, the issue becomes will there be enough serial isolation events in a given region for the cumulative sum total of divergence to eventually result in more speciation events ... the key condition stated for the MIM is that periods of isolation are shorter than the time to speciation, .. it is essentially the standard allopatric secondary contact model, just replayed multiple times.

Thus, if the MIM is stripped of this condition, no one would argue more species will be produced in cases where they become geographically isolated more often for periods of time sufficient for them to speciate.

Reply #5:

The reviewer has missed the many advantages of the MIM model over the allopatric model of (nearly) permanent isolation. The MIM mechanism has 4 advantages: i) Many more geographical features can satisfy the requirements of MIM. ii) The migration phase can help the dispersal of new species when speciation is complete. The two points (i and ii) are mutually complementary. Many geographical features do not permit isolation long enough for speciation to complete. Examples include many features associated with the glaciations. On the other hand, while permanent barriers like the Isthmus of Panama are conducive for speciation, they also prohibit dispersal after speciation. Hence, the species number would double but continual speciation does not happen. The last paragraph of the main text states

> If speciation is achieved after j cycles, then the number of species after n cycles would be $[i]^{n/j}$. In other words, the number of species after n cycles can potentially be m^n where m = $i^{1/j}$ > 1. In the special case of i = 2 and j =1, m = 2^n. Centers of high biodiversity are fascinating phenomena with many possible causes (20-22, 79, 80). We suggest that efficient speciation mechanisms like MIM cycles may play a role.

There are additional advantages for speciation via the MIM cycles: iii) Reproductive incompatibility is more likely to evolve if there are phases of strict isolation (even though these phases are interspersed with phases of concentrated gene flow). iv) Phases of isolation may even speed up adaptive divergence. A paragraph of Discussion is reproduced here.

> Interestingly, it has been posited that gene flow may even speed up speciation (the blue dotted line in Fig. 4e). This could happen if and when adaptive gene complexes, built up during isolation, are shuffled to generate many new combinations. Hybrid speciation (65-67) and adaptive radiation by hybrid swarms are such examples (68). Furthermore, many domesticated breeds were indeed created by hybridization between existing varieties (69-71). Thus, both plant and animal domestication resembles the MIM cycles, whereby breeds were separately domesticated with occasional exchange of genes. Although the idea of well-timed gene flow speeding up speciation is attractive, there is currently no evidence that it applies to mangrove speciation.

6) There are additional criticisms, which, not central to the study, are the reviewer's view of the genetic mechanisms of speciation.

7) Point 7 is from another reviewer.

The paper ... has an odd focus on the Strait of Malacca, a narrow stretch of a few hundred miles. But the species pairs that are studied occur over ranges that extend for many thousands of miles.

Reply #7:

For some reasons, this criticism happens more than once even though Fig. 1 clearly marks our sampling sites stretching from Hainan Island off the coast of China to the west coast of Thailand, a distance of several thousand miles, covering the main growing areas of these species. The problem may be that most people study terrestrial organisms. So, they tend to think that a strait (like the Strait of Malacca) is a barrier when the sea level is high. For mangroves, seed migration is via ocean currents and the strait is in fact a conduit of migration. It becomes a barrier only when the sea level is low. During glaciations, the shallow sea floor (marked by light blue in Fig. 1a) is dry land; hence, migration between the east and west coasts of Thailand is entirely blocked.

Estimation of substitution rates

We used an average substitution rate 4.78×10^{-9} /site/year previously estimated for the internal transcribed spacers (ITS, including the 5.8S gene) of the nuclear ribosomal DNA (77) as the nucleotide substitution rate in the genera *Lumnitzera* and *Xylocarpus*.

For the other seven genera (*Rhizophora*, *Bruguiera*, *Ceriops*, *Kandelia*, *Sonneratia*, *Avicennia* and *Aegiceras*), we collected exome/transcriptome data from a previous study (ref. (26) and He *et al*., unpublished data) and acquired putative orthologs using OrthoMCL (93). We reconstructed phylogenetic trees for these lineages using PhyML (91) and estimated species divergence time using the HKY85+gamma model and the independent rate for each branch using *mcmctree* (92) with fossil dating (ref. (26) and He *et al*., unpublished data).

Because the substitution rates μ_{exon} above are estimated from CDS (Coding DNA Sequence), we adjusted the substitution rates μ of genomic DNA using the following method. We approximately inferred μ_{intron}/μ_{exon} using

K_{intron}/K_{exon}, where K is the number of segregating sites between two sequences from sibling species. Hence, the substitution rate μ of the whole genome is weighted by the length of introns and exons (Table S6).

Statistical tests of the geographical modes of speciation

We used two methods to test for the existence of gene flow during the speciation process between the two *Rhizophora* species, *R. mucronata* and *R. stylosa (*Fig. S2; Table S3). The first method uses the contrasting patterns of variance of divergence levels among genic vs. intergenic sequences (46). The genic sequences span the regions flanking both the 5' and 3' ends of the protein coding sequences, while the intergenic sequences covering the other regions. We removed 1 Kb from the head and tail from each intergenic regions in the following analysis. Since genic regions are more likely to be involved in ecological adaptation in the early stage of speciation, they may exhibit increased among-locus variance. The second method uses only intergenic regions and tests for a constant or variable species divergence time across the genome. Both methods implement a likelihood-ratio test framework and allow comparisons of an allopatric model (H_0) and a speciation-with-gene-flow model (H_1). The likelihood-ratio test results of both methods showed that the null model, i.e., allopatry model could be rejected at a significance level of 0.001, hence suggesting the existence of gene flow during the speciation process between *R. mucronata* and *R. stylosa*.

Genome-wide alignments including both genic and intergenic regions of two nascent species were required to use the method developed by Osada and Wu (46). We mapped *R. mucronata* and *R. stylosa* sequence reads to the genome of *R. apiculata* separately and extracted consensus genome sequences. We only retained genomic sites that had a mapping depth no less than three. The nucleotide with the higher read count was retained at heterozygous sites. If the read count was equal we randomly selected between the alternatives. After obtaining the two consensus genomes and their alignment, we extracted genic region sequences and retained 7,771 whose lengths were larger than 300 bp. Using these 7,771 sequence length values, we sampled 7,771 sequences from the intergenic regions (IGS) while keeping the divergence level under 5%.

We counted the number of mismatches for each intergenic sequence and used the program KaKs_Calculator (94) to calculate the number of synonymous substitutions for the 7,771 genic region sequences. The sequence length and number of substitutions were used in the maximum-likelihood estimation procedure for divergence time and ancestral population size, as well as the log-likelihood value.

To use the method implemented in ref. (47), a genome alignment of three species was required. Consensus genomes of *R. mucronata* and *R. stylosa* were extracted as described above, and aligned together with the *R. apiculata* genome. The sequences remaining after masking genic regions were considered to be intergenic. We removed 1 Kb from the head and tail from each intergenic region and retained sequences with more than 2 Kb of data present in all three species. The final data set containing 9,745 sequences from intergenic regions was fed to the "3s" program (47).

Demographic model construction and parameter estimation

Because the Strait of Malacca has opened and closed many times due to fluctuating sea levels during glacial periods over the past millions of years (Fig. 1b), we suggest an MIM model to describe this multiple isolation and mixing pattern (Fig. 3c). When populations are isolated during the I (isolation) phase, migration between populations is blocked. When the Strait of Malacca opens during the M (mixing) phase, the potential for migration is restored. These migration/isolation cycles repeated multiple times during the time considered in this study. The MIM model is constructed following real geological events.

For comparison, we also constructed a model with a single isolation phase between two demes (SIM, Fig. 3a). We used a maximum likelihood method to estimate $N_e\mu$ and N_em (N_e: effective population size of a single deme; μ:

mutation rate; m: migration rate from one deme to another). We assumed the migration rate to be symmetric between pairs of populations.

The number of different nucleotides between two sequences sampled from the same population was denoted D_w, while that between different populations was denoted D_b (the subscript w is for within population and b for between populations). Therefore, the log-likelihood function can be constructed as follows:

$$\begin{aligned} Log(L) &= Log(\prod_x P(D_b = x)^{f(D_b=x)} \prod_y P(D_w = y)^{f(D_w=y)}) \\ &= \sum_x f(D_b = x) Log(P(D_b = x)) + \sum_y f(D_w = y) Log(P(D_w = y)) \end{aligned} \text{. Eq. (S1)}$$

$f(D_b = x)$ is the observed number of sequence pairs between populations in which D_b is equal to x and $f(D_w = y)$ is the observed number of sequence pairs within the population in which D_w is equal to y. The following is the derivation process of the probability of observing D_b equal to x ($P(D_b = x)$) and the probability of observing D_w equal to y ($P(D_w = y)$).

We used (2, 0) and (1, 1) to denote the state of two sequences in the same population and different populations, respectively. We used (1, 0) to denote the state of two sequences that coalesced.

In the coalescent process of a simple continual migration model (a single M phase lasting forever), the one-step transition probability matrix A is

$$A = \begin{array}{c} \\ (2,0) \\ (1,1) \\ (1,0) \end{array} \begin{array}{c} \begin{array}{ccc} (2,0) & (1,1) & (1,0) \end{array} \\ \begin{bmatrix} 1-2m-1/2N_e & 2m & 1/2N_e \\ 2m & 1-2m & 0 \\ 0 & 0 & 1 \end{bmatrix} \end{array} \text{. Eq. (S2)}$$

$A[1,3]$ (from (2, 0) to (1,0)) is $1/2N_e$, according to the classical coalescent theory. $A[2,3]$ is 0 because two sequences cannot coalesce when they are in different populations. After t generations, the t-step transition probability matrix is A^t . Therefore, the probability that (2, 0) has coalesced in the t-th generation is $A^t[1,3] - A^{t-1}[1,3]$.

The probability of a mutation occurring in one generation is 2μ, where μ is the mutation rate per generation. The number of mutations x occurring during t generations is binomially distributed.

Therefore, the probability of D_b equal to x is

$$P(D_b = x) = \sum_{t=1}^{\infty} (A^t[2,3] - A^{t-1}[2,3]) C_t^x (1-2\mu)^{t-x} (2\mu)^x \text{, Eq. (S3)}$$

where the term $C_t^x (1-2\mu)^{t-x} (2\mu)^x$ is the binomial probability of observing x mutations in t generations. Similarly, the probability that D_w is equal to y is

$$P(D_w = y) = \sum_{t=1}^{\infty} (A^t[1,3] - A^{t-1}[1,3]) C_t^y (1-2\mu)^{t-y} (2\mu)^y \text{ . Eq. (S4)}$$

The MIM model is more complex than a continual migration model. In the MIM model, the migration rate changes during different geological time periods. In the M phase, the migration rate is m. In the I phase, the migration rate is zero. We assume that each M lasts for k generations and that each I lasts for j generations. In the coalescent process during the M period the one-step transition probability matrix is A as in the simple migration model. However, in the I period the matrix is different because of the absence of migration:

$$B = \begin{array}{c} \\ (2,0) \\ (1,1) \\ (1,0) \end{array} \begin{array}{c} \begin{array}{ccc} (2,0) & (1,1) & (1,0) \end{array} \\ \begin{bmatrix} 1-1/2N_e & 0 & 1/2N_e \\ 0 & 1 & 0 \\ 0 & 0 & 1 \end{bmatrix} \end{array} \text{ . Eq. (S5)}$$

Assume the population is at the end of an M period now. The coalescence time for (2, 0) is $g(k+j)+t$ generations ($g \ge 0, 1 \le t \le k$, coalescence occurs in the M period) or $g(k+j)+k+t$ generations ($g \ge 0, 1 \le t \le j$, coalescence occurs in the I period).

Therefore, the probability that D_b is equal to x in the MIM model is

$$\begin{aligned} P(D_b = x) = \sum_{g=0}^{\infty} (\sum_{t=1}^{k} ((A^k B^j)^g A^t - (A^k B^j)^g A^{t-1})[2,3] C_{g(k+j)+t}^x (1-2\mu)^{g(k+j)+t-x} (2\mu)^x \\ + \sum_{t=1}^{j} ((A^k B^j)^g A^k B^t - (A^k B^j)^g A^k B^{t-1})[2,3] C_{g(k+j)+k+t}^x (1-2\mu)^{g(k+j)+k+t-x} (2\mu)^x) \end{aligned} \text{ . Eq. (S6)}$$

Similarly, the probability that D_w is equal to y in the MIM model is

$$\begin{aligned} P(D_w = y) = \sum_{g=0}^{\infty} (\sum_{t=1}^{k} ((A^k B^j)^g A^t - (A^k B^j)^g A^{t-1})[1,3] C_{g(k+j)+t}^y (1-2\mu)^{g(k+j)+t-y} (2\mu)^y \\ + \sum_{t=1}^{j} ((A^k B^j)^g A^k B^t - (A^k B^j)^g A^k B^{t-1})[1,3] C_{g(k+j)+k+t}^y (1-2\mu)^{g(k+j)+k+t-y} (2\mu)^y) \end{aligned} \text{ . Eq. (S7)}$$

In the SIM model, there is only one mixing phase (k generations) and one isolation phase (j generations). If two sequences did not coalesce in the recent M or I phase (the probability is $1-(A^k B^j)[1,3]$ for two sequences sampled from the same population and $1-(A^k B^j)[2,3]$ for two sequences sampled from different populations), they will coalesce in the single panmictic population with the probability $1/2N_e$ per generation. Therefore, the probability that D_b is equal to x in the SIM model is

$$\begin{aligned} P(D_b = x) = \sum_{t=1}^{k} (A^t - A^{t-1})[2,3] C_t^x (1-2\mu)^{t-x} (2\mu)^x + \sum_{t=1}^{j} (A^k B^t - A^k B^{t-1})[1,3] C_{k+t}^x (1-2\mu)^{k+t-x} (2\mu)^x \\ + (1-(A^k B^j)[2,3]) \sum_{t=1}^{\infty} c(1-c)^{t-1} C_{k+j+t}^x (1-2\mu)^{k+j+t-x} (2\mu)^x \end{aligned} \text{ . Eq. (S8)}$$

The probability that D_w is equal to y in the SIM model is

$$P(D_w = y) = \sum_{t=1}^{k}(A^t - A^{t-1})[1,3]C_t^y(1-2\mu)^{t-y}(2\mu)^y + \sum_{t=1}^{j}(A^kB^t - A^kB^{t-1})[2,3]C_{k+t}^y(1-2\mu)^{k+t-y}(2\mu)^y + (1-(A^kB^j)[1,3])\sum_{t=1}^{\infty}c(1-c)^{t-1}C_{k+j+t}^y(1-2\mu)^{k+j+t-y}(2\mu)^y \quad \text{Eq. (S9)}$$

We wrote *Mathematica* scripts to obtain maximum likelihood estimates of N_e and m for MIM and SIM models using numerical methods. The SIM model requires an additional parameter j, the isolation time imposed by the geographical isolation, as depicted in Fig. 3a. The time elements in the MIM model are defined by the geological records of sea level changes. Given a generation time equal to 20 years, the parameters j and k of MIM model were set to be 5,000 and 500 generations for each I and M phase according to geographical evidence of the recent cycles. The mutation rate is obtained from the fossil records as described in Table 1.

Adaptive genomic differentiation between geographical populations

In the incipient stage of speciation, divergent gene complexes may build up linkage block by linkage block (6, 12, 95-97). However, geographical populations without adaptive differentiation will not show such patterns. As a result, diverging geographical populations may be experiencing reduced gene flow due to selection against migrants and gene introgressions. The geographical distributions of mangroves are indeed uncorrelated with dispersal but are dependent on adaptive traits (98). Furthermore, some degree of adaptive divergence is common for plant populations with a mean F_{ST} of 0.3 or greater (ref. (99); see Fig. S16a-b).

The spread of the western haplotypes to the east and the eastern haplotypes to the west is given in Fig. S16a-b. Since only haplotypes that are present on both coasts are considered (see legends), the pattern reflects an increase in frequency after migration. The species that has the lowest degree of differentiation is *C. tagal (*only 9.3% East-West divergence as opposed to 43% - 73% in three other species shown) but this species also has proportionally the most haplotypes (blue bars) introgressed across the boundary. A particularly interesting case is *S. alba* which has intermediate diversity but exhibits the lowest level of gene flow from the West to the East. Interestingly, it also shows the highest level of gene flow in the reverse direction (Fig. S16b). Either the Western haplotypes are selected against in the East or many Eastern haplotypes are selected for in the West. At the most divergent stage is *Ae. corniculatum*, which has become differentiated within the East region (see Fig. S5c). There is little genetic mixing between the Gulf and Hainan in this species (Fig. S16c) although its dispersal rate is not low (100). Since the geographical barrier could not have been high between G and H as suggested by all other species, Fig. S16c may be explained by selection against migrants within the East region in *Ae. corniculatum*.

The application of PSMC to the SIM vs. MIM models

PSMC uses sequence divergence to gauge the effective population size (N_e). Hence, the temporal pattern of Ne displayed by PSMC is based on the distribution of the genetic diversity across loci. At the simplest level, the genetic diversity of a locus is expressed as $4N_e\mu/ (4N_e\mu+ 1)$. The PSMC method uses the whole genome data and assumes that each locus (or, more accurately, each linkage block) represents an independent demographical scenario in terms of population growth, sub-division, gene flow among populations etc.

Here, we use N_e to reflect the changes in the population subdivision. When two populations have diverged for a period of time the total diversity is determined more by the divergence time than by the actual number of individuals. (For example, if we have one genome from a human and one from the chimpanzee, the combine genetic diversity would suggest an effective size that is 20 times larger than the diversity within each taxa.)

In Fig. 4d, simulations of the SIM model yield a steep increase in Ne because the genetic diversity across all

loci started to increase at about the same time (see Fig. 3a). This shared demographic history gives rise to a fairly uniform distribution in the level of genetic diversity.

In contrast, under the MIM model, the level of genetic diversity may start to increase only in the most recent isolation phase when they began to diverge. This timing, as shown in Fig. 3c, is spread over the whole period, thus leading to a broader distribution of N_e in time. This broader distribution is closer to the observed Ne patterns displayed by PSMC (see Fig. 4d and Fig. S17).

Speciation model discussion

The MIM model postulates phases of migration and isolation. Are they both needed? In the genic view [(8)], migration is tolerated. In this section, we will expand on a theme that periodic isolation would help the evolution of reproductive isolation.

Gene flow breaks up genomes into islands with various levels of divergence, depending on the fitness of gene introgressions. Many studies have aimed at identifying the genomic "islands of speciation and divergence" (ISD) (14, 101-105). Such attempts are, however, fraught with uncertainties (15) because the statistical power for detecting ISD diminishes rapidly with time and with distance from the locus of hybrid incompatibility (106). To fully address the issue of “speciation with gene flow”, it would be necessary to combine a detailed knowledge of biogeography with an extensive genetic analysis.

An important aspect of speciation is the evolution of reproductive isolation (RI). This central aspect of speciation is where geographical isolation may be most crucial. If diverging populations are differentially adapted to the environments of different geographical regions, migration may not disrupt divergence, as pointed out by Wu (6) in connection with the genic view of speciation. This scenario is equivalent to the ecological speciation models (53, 54, 107). The evolution of RI, however, is very sensitive to gene flow as illustrated by the simplest Dobzhansky-Muller (DM) model below.

Consider a haploid two-locus model in which the two diverging populations start with the same (a, b) genotype. The genotype (a, b) can evolve to (A, b) or (a, B) and all three genotypes have the same fitness. (A, B) is inviable indicating that A and B are incompatible. The evolution of RI is complete when the two populations evolve to (A, b) and (a, B), respectively. When the two populations are in complete isolation, each population would evolve independently and there is a 50% chance of divergent evolution and, hence, RI. However, when there is migration before A and B are fixed in the two respective populations, their evolution is mutually interfering. In other words, when the two populations are in the process of fixing A and B, they would mutually slow down each other’s process of fixation through migration. In a deterministic model, RI would therefore not be able to evolve. Nevertheless, there is a non-zero probability of evolving RI in a stochastic framework when the population sizes are small. The dynamics are the same as in the problem of gene loss after duplication (108-110).

Since the interference would not happen when the two populations are in isolation, we ask whether periods of isolation in the process would help the evolution of RI in small populations. We compare two models – a constant migration (CM) model vs. the MIM model. In the two models, the total amount of migration over the entire period of speciation is the same. The only difference is that migration is distributed evenly across time in the CM model but is concentrated in the M phases in the MIM model.

In Fig. S18, we compare the probability of evolving RI by computer simulations for various $N\mu$ and Nm where N is the population size, m is the migration rate and μ is the mutation rate. As expected, when Nm increases, the probability of RI decreases. However, the rate of decrease is much lower under the MIM model than under the CM model, giving rise to a range of Nm values where RI is still highly likely under MIM but quite improbable under the CM model.

Since the genetics of hybrid incompatibility is rarely the simple 2-locus DM model used above (111), we simulated a multi-locus model. In multi-locus incompatibility, alleles from m loci of one species and n loci of the other interact to cause incompatibility, where m>1 and/or n>1. The simplest model has m=1 and n=2 in which the genotype (A, B, C) causes lethality. Fig. S19 presents the evolution of RI under a 3 (1+2) locus model. Here, the evolution of RI is also more likely under the MIM model than under the CM model. Furthermore, the more complex the genetics of RI, the greater the differences between MIM and CM in the probability of RI.

In a separate study (Yang *et al*., unpublished data), we model the evolution of RI more extensively considering the non-neutrality of fixation, the complex genetic interactions underlying RI and the durations of the M and I phases in the MIM model.

Table S1. Sampling locations and sample size

Species	Region	Stands	Coordinates	Salinity (‰)	Sample size
Ceriops tagal	Hainan Island	Wenchang	19°36'N, 110°47'E	9.3-12.6	100
		Yalong Bay	18°13'N, 109°36'E	3.2-25	100
	Gulf of Thailand	Tha Mai	12°23'N, 102°27'E	--	100
	West Thailand	Ngao	09°52'N, 98°36'E	~15	100
Rhizophora apiculata	Hainan Island	Wenchang	19°36'N, 110°47'E	9.3-12.6	30
		Tielu harbor	18°15'N, 109°42'E	25.8-32.3	30
		Sanya River	18°14'N, 109°30'E	9.2-30	30
	Gulf of Thailand	Chaiya	09°22'N, 99°15'E	29.3	34
		Khanom River	09°13'N, 99°49'E	4-10	34
	West Thailand	Ngao	09°52'N, 98°36'E	~17	34
Avicennia marina	Hainan Island	Wenchang	19°36'N, 110°47'E	9.3-29.6	100
		Sanya	18°15'N, 109°30'E	3.3-32.3	100
	Gulf of Thailand	Bang Ya Phraek	13°30'N, 100°16'E	2.4-7.0	19
		Bang Khun Sai	13°10'N, 100°01'E	~25	35
		Thongnian Bay	09°18'N, 99°48'E	19-29	35
	West Thailand	Leamson	09°35'N, 98°29'E	~24.3	35
Sonneratia alba	Hainan Island	Qionghai	19°13'N, 110°36'E	6.8-18.7	100
		Yalong Bay	18°13'N, 109°36'E	3.2-25	85
	Gulf of Thailand	Chaiya	09°22'N, 99°15'E	29.3	50
		Thongnian Bay	09°18'N, 99°48'E	19-29	50
	West Thailand	La-Un	10°10'N, 98°43'E	3.5	50
		Ngao	09°52'N, 98°36'E	~17	50
		Leamson	09°35'N, 98°29'E	~24.3	50
Aegiceras corniculatum	Hainan Island	Wenchang	19°36'N, 110°47'E	9.3-12.6	100
		Yalong Bay	18°13'N, 109°36'E	3.2-25	50
		Sanya River	18°14'N, 109°30'E	9.2-30	24
	Gulf of Thailand	Chaiya	9°23'N, 99°16'E	29.3	50
	West Thailand	La-Un	10°10'N, 98°43'E	3.5	36
		Ngao	09°52'N, 98°36'E	~17	51

Table S2. Genes used in the population sequencing of the five species

**Gene ID**	**GenBank Accession No.**	**Length (bp)**	**Exon region Length (bp)**
Ceriops tagal			
CTP001	JQ794711	1057	0
CTP002	JQ794659	699	132
CTP003	JQ794746	2332	917
CTP004	JQ794660	1297	351
CTP005	JQ794747	1258	258
CTP006	JQ794661	961	903
CTP007	JQ794662	432	372
CTP008	JQ794663	520	243
CTP009	JQ794664	1037	222
CTP010	JQ794665	837	201
CTP011	JQ794666	924	249
CTP012	JQ794667	515	313
CTP013	JQ794668	1665	612
CTP014	JQ794669	648	159
CTP015	JQ794670	1579	207
CTP016	JQ794671	868	291
CTP017	JQ794672	878	147
CTP018	JQ794673	781	159
CTP019	JQ794674	583	264
CTP020	JQ794675	676	297
CTP021	JQ794676	983	312
CTP022	JQ794677	1049	147
CTP023	JQ794678	466	456
CTP024	JQ794679	945	228
CTP025	JQ794680	1402	93
CTP026	JQ794748	1256	240
CTP027	JQ794681	564	402
CTP028	JQ794682	1819	345
CTP029	JQ794683	613	156
CTP030	JQ794684	1445	177
CTP031	JQ794685	478	147
CTP032	JQ794686	1638	180
CTP033	JQ794687	829	102
CTP034	JQ794688	840	270

CTP035	JQ794689	1363	210
CTP036	JQ794690	1391	213
CTP037	JQ794691	1196	108
CTP038	JQ794692	1677	285
CTP039	JQ794693	879	225
CTP040	JQ794694	653	150
CTP041	JQ794695	515	258
CTP042	JQ794696	704	270
CTP043	JQ794697	602	84
CTP044	JQ794698	753	270
CTP045	JQ794699	916	165
CTP046	JQ794700	511	212
CTP047	JQ794701	513	501
CTP048	JQ794702	754	83
CTP049	JQ794703	910	0
CTP051	JQ794705	868	162
CTP052	JQ794706	505	252
CTP053	JQ794707	569	93
CTP054	JQ794708	957	156
CTP055	JQ794709	725	120
CTP056	JQ794710	866	138
CTP057	JQ794712	650	402
CTP058	JQ794713	1812	1037
CTP059	JQ794714	1391	591
CTP060	JQ794715	773	294
CTP061	JQ794716	1037	282
CTP062	JQ794717	1197	468
CTP063	JQ794718	1018	966
CTP064	JQ794719	1064	372
CTP065	JQ794720	2256	186
CTP066	JQ794721	1439	543
CTP067	JQ794722	1023	186
CTP068	JQ794723	1075	289
CTP069	JQ794724	1116	180
CTP070	JQ794725	1976	162
CTP071	JQ794726	887	342
CTP072	JQ794727	455	198
CTP073	JQ794728	1108	369
CTP074	JQ794729	2207	276
CTP075	JQ794730	757	135

CTP076	JQ794731	675	302
CTP077	JQ794732	600	150
CTP078	JQ794733	845	291
CTP079	JQ794734	721	324
CTP080	JQ794735	671	363
CTP081	JQ794736	1099	315
CTP082	JQ794737	472	288
CTP083	JQ794738	314	288
CTP084	JQ794739	494	282
CTP085	JQ794740	900	213
CTP086	JQ794741	1355	114
CTP087	JQ794742	908	229
CTP088	JQ794743	381	165
CTP089	JQ794744	581	177
CTP090	JQ794745	927	171
Avicennia marina			
Am0008	KC928137	891	206
Am0009a	KC928138	879	462
Am0038	KC928139	1867	389
Am0054	KC928140	1768	297
Am0055	KC928141	1794	269
Am0056a	KC928142	884	500
Am0066	KC928143	875	296
Am0069	KC928144	709	457
Am0072	KC928145	510	414
Am0073	KC928146	1262	529
Am0094	KC928147	1433	167
Am0096	KC928148	599	126
Am0100	KC928149	959	124
Am0101	KC928150	959	302
Am0115	KC928151	949	129
Am0118	KC928152	531	91
Am0123	KC928153	490	244
Am0129b	KC928154	768	155
Am0154	KC928155	692	246
Am0156	KC928156	905	290
Am0182	KC928157	655	289
Am0187	KC928158	801	321
Am0190	KC928159	1414	180
Am0197	KC928160	567	210

Am0201	KC928161	1897	289
Am0218	KC928162	508	209
Am0219	KC928163	1022	231
Am0226	KC928164	931	338
Am0229	KC928165	605	158
Am0245	KC928166	406	175
Am0251	KC928167	1709	262
Am0256	KC928168	1657	144
Am0257	KC928169	775	104
Am0258	KC928170	879	187
Am0259	KC928171	847	202
Am0269	KF918415	960	0
Am0278	KC928172	2849	274
Am0279	KC928173	540	129
Am0284	KC928174	626	137
Am0287	KC928175	2575	85
Am0291	KC928176	1107	264
Am0293	KC928177	1014	184
Am0323	KC928178	1271	246
Am0324	KC928179	1653	355
Am0326	KC928180	729	285
Am0327	KC928181	418	327
Am0332	KC928182	420	295
Am0333	KC928183	1406	245
Am0339	KC928184	1723	327
Am0341	KC928185	1093	123
Am0347	KC928186	738	177
Am0349a	KC928187	702	143
Am0363	KC928188	1144	173
Am0365	KC928189	1038	44
Amc006	KC928190	597	300
Amc007	KC928191	359	190
Amc018b	KC928192	940	256
Amc019	KC928193	759	343
Amc037	KC928194	1699	202
Amc038	KC928195	1030	265
Amc052	KC928196	450	242
Amc099	KC928197	627	204
Amc119	KC954607	1352	198
Amc121	KC928198	1165	193

Amc129	KC928199	729	226
Amc136	KC928200	838	260
Amc137	KC928201	697	166
Amc138	KC928202	1125	213
Amc171	KC928203	748	206
Amc201	KC928204	2716	137
Amc202	KC928205	837	309
Amc208	KC928206	1069	373
Amc213	KC928207	656	103
Amc214	KC928208	907	363
Amc221	KC928209	756	217
Amc231	KC928210	518	172
Amc232	KC928211	654	132
Amc233	KC928212	598	301
Amc244	KC928213	882	277
Amc246	KC928214	1292	340
Amc253	KF918414	495	332
Amc266	KC928215	534	209
Amc270	KC928216	429	85
Amc273	KC928217	1727	85
Amc285	KC928218	855	364
Amc299	KC928219	735	231
Amc302	KC928220	1195	157
Amc309	KC928221	533	133
Amc314	KC928222	1347	251
Amc320	KC928223	942	78
Amc324	KC928224	951	302
Amc348	KC928225	549	265
Amc351	KC928226	1965	277
Amc361	KC928227	1428	318
Amc362	KC928228	920	154
Sonneratia alba			
Sa1	GQ121993.1	730	272
Sa4	GQ121994.1	1678	643
Sa10	GQ121995.1	1572	232
Sa37	GQ121996.1	1218	556
Sa38	GQ121997.1	765	227
Sa59	GQ121998.1	1392	205
Sa64	GQ121999.1	1097	342
Sa66	GQ122000.1	1127	389

Sa80	GQ122001.1	645	187
Sa82	GQ122002.1	814	270
Sa90	GQ122003.1	942	512
Sa91	GQ122004.1	1099	247
Sa100	GQ122006.1	1083	332
Sa164	GQ122007.1	869	169
Sa165	GQ122008.1	824	346
Sa174	GQ122009.1	566	322
Sa236	GQ122011.1	766	266
Sa251	GQ122013.1	1162	467
Sa259	GQ122014.1	2093	346
Sa269	GQ122015.1	663	315
Sa281	GQ122016.1	1503	142
Sa308	GQ122017.1	968	242
Sa343	GQ122020.1	1418	250
Sa352	GQ122021.1	770	337
Sa361	GQ122022.1	733	150
Sa369	GQ122023.1	1089	237
Sa370	GQ122024.1	748	233
Sa389	GQ122027.1	1585	324
Sa393	GQ122028.1	1496	273
Sa402	GQ122029.1	1539	385
Sa423	GQ122030.1	796	310
Sa431	GQ122031.1	753	216
Sa431	GQ122031.1	753	216
Sa449	GQ122032.1	1499	267
Sa465	GQ122033.1	1715	245
Sa473	GQ122034.1	1857	88
Sa474	GQ122035.1	1133	181
Sa475	GQ122036.1	909	351
Sa476	GQ122037.1	1074	350
Sa480	GQ122038.1	907	520
Sa496	GQ122039.1	860	170
Sa519	GQ122040.1	774	167
Sa526	GQ122041.1	843	306
Sa529	GQ122042.1	1020	343
Sa530	GQ122043.1	1125	350
Sabu4	GQ122044.1	614	322
Sabu36	GQ122046.1	665	248
Sabu40	GQ122047.1	959	232

Sabu56	GQ122048.1	1401	409
Sabu93	GQ122049.1	1001	416
Sabu110	GQ122050.1	1312	264
Sabu120	GQ122051.1	1198	413
Sanhx2	GQ122052.1	1114	633
Saxx17	GQ122054.1	886	0
Sayy09	GQ122055.1	1655	348
Sayy22	GQ122057.1	1162	552
Sayy31	GQ122058.1	1836	406
Sayy39	GQ122059.1	1288	323
Sayy42	GQ122060.1	1164	387
Sayy51	GQ122061.1	1114	467
Sayy65	GQ122062.1	1805	255
Sayy77	GQ122063.1	631	310
Aegiceras corniculatum			
A023	KF745073	1544	366
A029	KF745075	707	79
A041	KF745076	859	0
A047	KF745077	1181	263
A056	KF745078	1254	0
A086	KF745079	1075	94
A089	KF745080	804	249
A093	KF745081	852	0
A102	KF745082	869	348
A107	KF745083	480	352
A108	KF745084	632	307
A110	KF745085	1775	372
A115	KF745087	1232	261
A127	KF745088	1003	201
A132B	KF745090	861	0
A159	KF745092	843	0
A170	KF745095	551	100
A189R	KF745096	823	0
A194	KF745097	1251	255
A244	KF745099	596	120
A245-2	KF745100	1290	0
A271	KF745101	1337	0
A302	KF745102	1243	264
A304R	KF745103	424	223
A312	KF745104	771	422

A320	KF745105	1470	0
A336-2	KF745106	669	320
A348	KF745108	373	89
A356	KF745109	2422	252
A365	KF745110	1271	324
A366B	KF745111	808	170
A382	KF745113	1800	226
A383	KF745114	846	255
A386	KF745115	554	162
A414	KF745116	822	368
A440	KF745117	904	109
A449	KF745118	933	221
A458	KF745119	1663	249
A469	KF745120	1320	432
Ac258A	KF745121	1241	275
Ac258B	KF745122	951	260
Ac266A	KF745123	1274	394
Ac266B	KF745124	1181	0
Ac268	KF745125	961	203
C008	KF745127	606	147
C015	KF745128	747	299
C016	KF745129	1329	503
C019	KF745130	1167	363
C053	KF745132	783	0
C058	KF745133	530	283
C060A	KF745134	1312	93
C076-1	KF745135	621	271
C079-1	KF745136	542	139
C080	KF745137	508	301
C088	KF745138	921	310
C098	KF745139	1618	426
A208	KF958338	899	358
A249-1800	KF958340	1821	293
A431	KF958343	1453	524
C041	KF958344	749	0
C060B	KF958345	823	243
A070	KF977861	1130	0
A139B	KF977862	1392	0
A290	KF977863	402	0

A324A	KF977864	854	0
A438	KF977865	983	0
C096	KF977866	1602	0
Rhizophora apiculata			
22477	KF793837	810	159
22624	KF793838	466	150
22685	KF793839	576	228
23056	KF793840	413	165
sos2	KF793841	1579	474
23186	KF793842	900	183
18	KF793843	957	363
22	KF793844	809	570
22786	KF793845	1105	585
22874	KF793846	866	288
22454	KF793847	1529	441
22025	KF793848	588	264
c052	KF793849	971	180
C056	KF793850	909	906
c057	KF793851	503	102
c060	KF793852	788	333
c5	KF793853	723	75
c2	KF793854	934	300
c3	KF793855	692	126
c4	KF793856	669	300
C22	KF793857	703	309
22244	KF793858	1287	441
22292	KF793859	945	360
22066	KF793860	1455	483
C1	KF793861	1677	807
C18	KF793862	903	384
C26	KF793863	879	309
21993	KF793864	1276	486
c31	KF793865	784	198
CT10-L	KF793866	1090	102
CT36-3-R	KF793867	482	183
CT61	KF793868	876	201
CT64	KF793869	666	138
20	KF793870	526	351
22728	KF793871	961	306

CT55-1	KF793872	708	102
15979	KF793873	1298	390
22274	KF793874	1572	300
23474	KF793875	525	321
23426	KF793876	492	90
B130	KF793877	650	225
B28-1-R	KF793878	1409	39
23450-R	KF793879	709	225
23450-L	KF793879	568	189
B129-L	KF793880	625	144
B121-2	KF793881	837	156
C41	KF793882	992	231
23478	KF793883	1457	318
B3-2-R	KF793884	961	228
B113-R	KF793885	1671	339
23462	KF793887	1272	342
B132-2	KF793888	780	75
B119	KF793889	773	687
B21	KF793890	2085	360
C49	KF793891	1277	528
23416	KF793892	2148	171
23632	KF793893	609	168
23793	KF793894	1370	540
23852	KF793895	1699	639
23839	KF793896	1403	357
23800	KF793897	580	75
23752	KF793898	1164	534
23794	KF793899	260	258
23714	KF793900	1612	480
23683	KF793901	1984	516
23665	KF793902	1500	288
23770	KF793903	810	87
23636	KF793904	771	255
23691	KF793905	423	354
23777	KF793906	1830	384
23790	KF793907	743	345
DLDH	KF793908	1219	1086
PAL1	KF793909	938	936
SBE2	KF793910	1349	249
mang-1	KF793911	369	213

CT19-2-c10	KF793912	1645	984
23319-c045	KF793913	967	441
c081-2	KF933386	901	885

Table S3. ***R. mucronata*** **and** ***R. stylosa*** **likelihood ratio test results using two methods**.

**Method1: Osada & Wu (2005)**					
H_0: $\gamma_{GS} = \gamma_{IGS} = \gamma_0$[a]	γ_0	θ_{GS}	θ_{IGS}		ln *L*
	0.2277	0.0038	0.0052		-32486.8060
H_1: $\gamma_{GS} \neq \gamma_{IGS}$	γ_{GS}	γ_{IGS}	θ_{GS}	θ_{IGS}	ln *L*
	0.6764	0.0916	0.0027	0.0060	-32281.2133
P-value[b]					2.02×10^{-91}

**Method2: Yang (2010)**					
H_0	θ_0[c]	θ_1	τ_0	τ_1	ln *L*
	0.0110	0.0039	0.0067	0.0007	-1970390.58
H_1	θ_0	θ_1	τ_0	τ_1	ln *L*
	0.0111	0.0035	0.0067	0.0008	-1970190.10
P-value[b]					3.41×10^{-89}

a: $\gamma = \tau / \theta$. For H_0, this ratio was assumed to be the same between intergenic sequences (IGS) and genic sequences (GS) and θ was estimated for IGS and GS separately. For H_1, both τ and θ was estimated for IGS and CDS separately.

b: P values of both tests were obtained from the chi-squared distribution with one degree of freedom.

c: θ_0 denotes the ancestral population size for all three extant species and θ_1 denotes the ancestral population size for the two extant species that diverged later. τ_0 denotes the time interval between root and the internode and τ_1 denotes the divergence time for the latter two diverged species (see also Fig. S2).

Table S4. Nucleotide diversity for population stands (π_S) and regions (π_R)

Species	Region	π_S/Kb	E (π_S)/Kb[a]	π_R/Kb[a]
Ceriops tagal	H	0.248, 0.174	0.210	0.219
	G	0.308	--	--
	W	0.229	--	--
Rhizophora apiculata	H	0.315, 0.391, 0.373	0.360	0.451
	G	0.665, 0.557	0.611	0.586
	W	0.897	--	--
Sonneratia alba	H	0.000, 0.000	0.000	0.260
	G	0.469, 0.197	0.333	0.435
	W	1.290, 1.335, 1.095	1.240	1.408
Avicennia marina	H	0.221, 0.320	0.271	0.316
	G	0.923, 0.936, 0.805	0.888	0.934
	W	0.867	--	--
Aegiceras corniculatum	H	2.118, 1.601, 1.414	1.711	2.697
	G	1.224	--	--
	W	3.941, 3.474	3.707	4.000

H: Hainan; G: Gulf of Thailand; W: West Coast

a: Not calculated when only one stand within a region. Nucleotide diversity of a region is represented by that of the stand in that case.

Table S5. Sources of DNA sequences to estimate the genetic divergence among species

Genus; species	Gene Accession No.
Sonneratia	
S. caseolaris	See Zhou *et al*., 2007 (112)
S. lanceolata	KP256899, KP256922, KP256926, KP257048, KP256992, KP257015, KP257036, KP256954
S. alba	See Zhou *et al*., 2007 (112)
S. ovata	See Zhou *et al*., 2007 (112)
S. griffithii	See Yang *et al*., 2014 (113)
S. apetala	See Zhou *et al*., 2007 (112)
Kandelia	
K. obovata	KP699077, KP699078, KP699079
K. candel	KP699074, KP699075, KP699076
Bruguiera	
B. gymnorhiza	See Urashi *et al*., 2013 (114)
B. sexangula	See Urashi *et al*., 2013 (114)
Rhizophora	
R. apiculata	See Inomata *et al*., 2009 (115)
R. mucronata	See Inomata *et al*., 2009 (115)
R. stylosa	See Ng and Szmidt, 2015 (116)
Ceriops	
C. australis	See Huang *et al*., 2012 (117)
C. tagal	See Huang *et al*., 2012 (117)
C. decandra	KC854434, KC854457, KC854481, KC854505, KC854528, KP699059, KP699060, KP699061, KP699062, KP699063, KP699064, KP699065, KP699066, KP699067, KP699068
C. pseudodecandra	KC854426, KC854451, KC854473, KC854499, KC854522,
C. zippleana	KC854440, KC854463, KC854487, KC854511, KC854534,
Xylocarpus	
X. grantum	KP699080, KP699081, KP699082
X. moluccensis	KP699083, KP699084, KP699085
Aegiceras	
Ae. corniculatum	KF745096, KF745133, KF745116, KF745117
Ae. flordium	KP699069, KP699070, KP699072, KP699073
Lumnitzera	
L. racemosa	KF477443, KF477446, KF496922, KF477449, KF496924, KF477450, KF477452, KF477453, KF553913, KF553914, KF477455, KF477456, KF477457, KF477458, KF477459,

	KF477460, KF477465, KF477466, KF477467, KF477468, KF477472, KF477474, KF477475, KF477479, KF477488, KF477497, KF477502, KF477504,
L. littorea	KP661139, KP661140, KP661162, KP661141, KP661165, KP661142, KP661143, KP661144, KP661163, KP661164, KP661145, KP661146, KP661147, KP661148, KP661149, KP661150, KP661151, KP661166, KP661152, KP661153, KP661154, KP661155, KP661156, KP661157, KP661158, KP661159, KP661160, KP661161,
Avicennia	
Av. alba	KP025973, KP026059, KP025981, KP025989, KT453675, KP026013, KP026020, KP026027, KP026034, KP026041, KP026046, KP026052, KT453698, KP026072, KP026107, KP026113, KT453704, KT453713, KT453720, KT453686, KP026079, KT453691, KT453694
Av. rumphiana	KP025980, KP026065, KP025987, KP025995, KP026002, KP026012, KP026019, KP026026, KP026033, KP026040, KP026045, KP026051, KP026058, KT453699, KP026078, KP026119, KT453706, KT453712, KT453721, KP026071, KP026084, KP026092, KP026098, KP026106
Av. marina var. *marina*	KP025976, KP026062, KP025984, KP025992, KP025998, KP026007, KP026016, KP026024, KP026030, KP026037, KP026048, KP026055, KT453702, KP026075, KT453696, KT453697, KT453708, KT453710, KT453716, KT453688, KP026089, KP026095, KP026103
Av. marina var. *eucalyptifolia*	KP025975, KP026061, KP025983, KP025991, KT453674, KT453676, KT453678, KP026023, KT453680, KP026036, KP026047, KT453684, KT453700, KT453689, KP026109, KP026115, KT453707, KT453718, KT453687, KT453690, KT453692, KP026094, KT453695
Av. marina var. *australasica*	KP025977, KP026063, KP025985, KP025993, KP025999, KP026008, KP026017, KP026025, KP026031, KP026038, KP026043, KP026049, KP026056, KT453701, KP026076, KP026110, KP026116, KT453709, KT453711, KT453717, KP026082, KP026090, KP026096, KP026104
Av. officinalis	KT453671, KT453685, KT453672, KT453673, KP026000, KT453677, KT453679, KT453681, KT453682, KP026044, KT453683, KP026057, KT453703, KP026077, KP026112, KP026118, KT453705, KT453714, KT453719, KP026070, KP026083, KP026091, KT453693, KP026105

Av. integra	KT453655, KT453656, KT453657, KT453658, KT453659, KT453660, KT453661, KT453662, KT453663, KT453664, KT453665, KT453666, KT453667, KT453668, KT453669, KT453670

Table S6. Estimation of substitution rates for *Avicennia*, *Aegiceras*, *Sonneratia*, and *Rhizophoreae*

**Sibling species**	**μ_{exon}[a] (10^{-9}/site/year)**	**μ_{intron} / μ_{exon}[b]**	**μ_{intron} (10^{-9}/site/year)**	**$Length_{exon}$ (bp)**	**$Length_{intron}$ (bp)**	**μ_{total}[c] (10^{-9}/site/year)**
Av. marina vs. *Av. alba*	1.63	2.063	3.36	20,654	64,520	3.05
Ae. corniculatum vs. *Ae. flordium*	2.03	3.465	7.03	9,725	47,478	6.43
S. alba vs. *S. caseolaris*	1.42	2.133	3.03	14,406	45,205	2.81
B. gymnorhiza vs. *B. sexangula*	0.66	2.712	1.79	3,294	1,943	1.08
C. tagal vs. *C. decandra*	0.90	1.794	1.62	19,258	57,374	1.44
R. apiculata vs. *R. stylosa*	0.82	1.619	1.33	21,154	44,033	1.16
K. candel vs. *K. obovata*	0.81	3.000	2.43	1,324	1,762	1.74

a: μ_{exon} is estimated from coding sequence with fossil dating (ref. (26) and He *et al*., unpublished data).

b: $\mu_{intron}/\mu_{exon} = K_{intron}/K_{exon}$, where K is the number of segregating sites between two sequences of sibling species.

c: $\mu_{total} = (\mu_{exon} \times Length_{exon} + \mu_{intron} \times Length_{intron})/(Length_{exon} + Length_{intron})$.

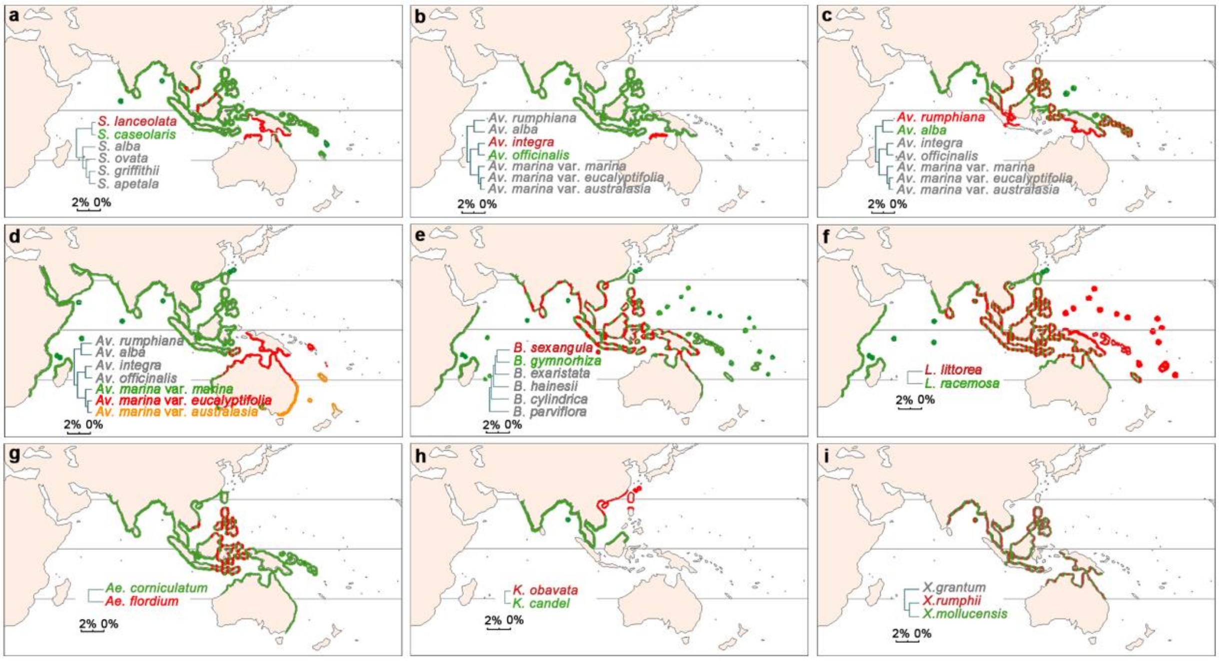

Figure S1. Distributions of closely-related mangrove species in IWP and its geography. (a) *Sonneratia*; (**b** to **d**) *Avicennia*; (**e**) *Bruguiera*; (**f**) *Lumnitzera*; (**g**) *Aegiceras*; (**h**) *Kandelia*; (**i**) *Xylocarpus*. Distribution of each species is redrawn based on *Mangrove ID (33)* . Species pairs geographically delineated by the Strait of Malacca are shown in red and green.

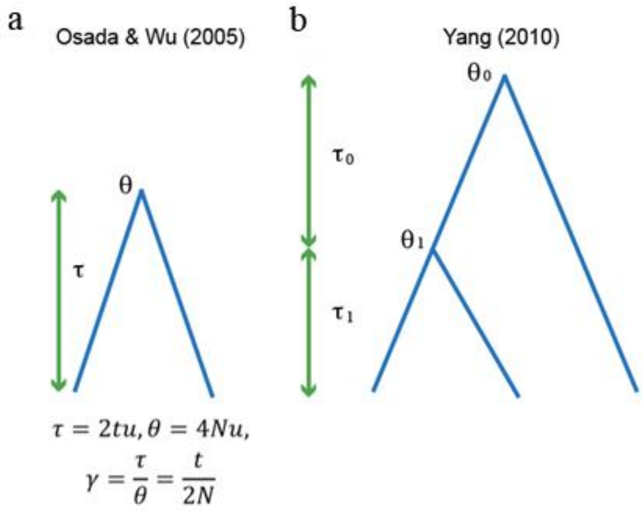

Figure S2. (a) The two-species tree used in Osada & Wu's method (46), there are two parameters in the model: one parameter characterizing the ancestral population size (θ), one parameter for divergence time (τ). The method also defines γ=τ/θ=t/2N and tests if γ is the same between the genic and intergenic regions. (**b**) The three-species tree used in Yang (2010)'s method (47), there are four parameters in the model: two parameters characterizing ancestral population size (θ_0 and θ_1), two parameters for divergence time (τ_0 and τ_1). This method relies on the variance in divergence across the genome, taking incomplete lineage sorting as four possible different gene trees into account.

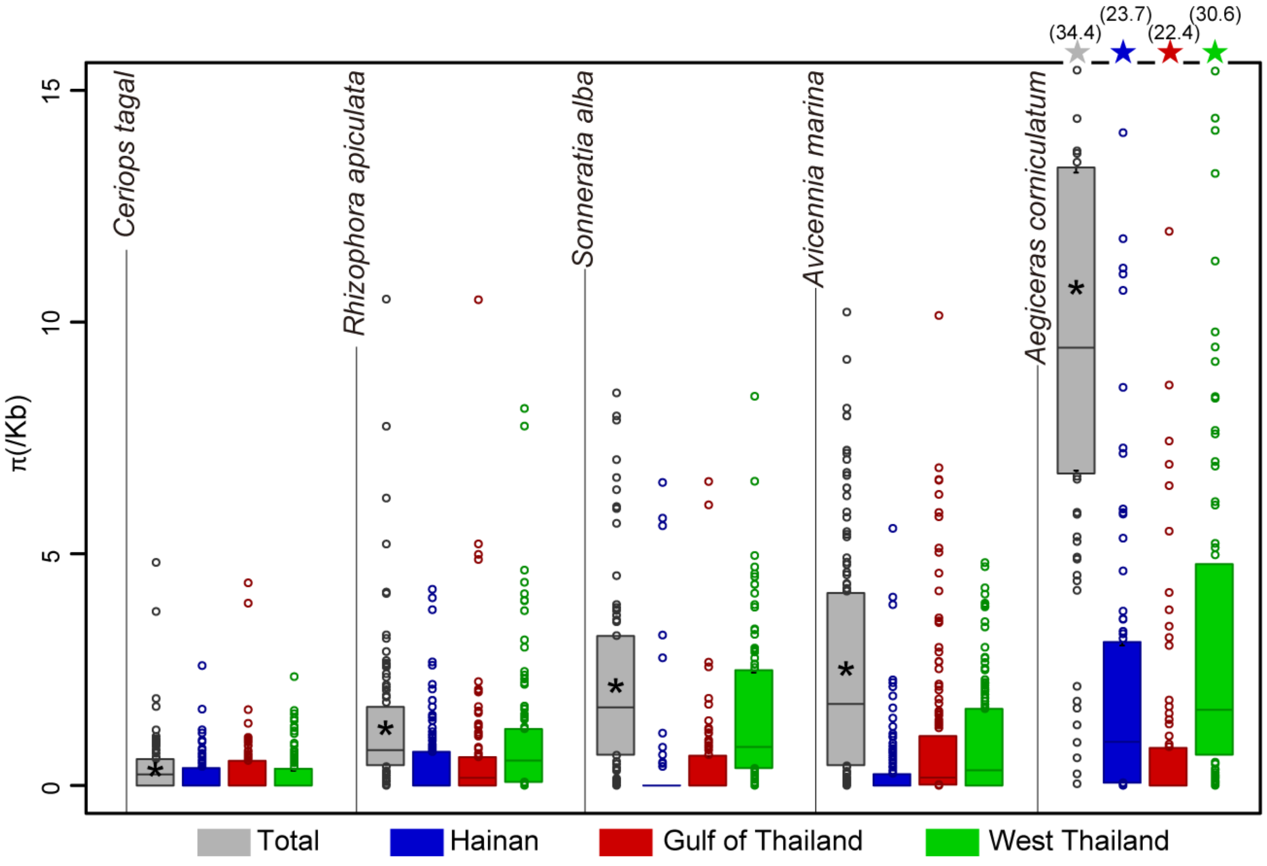

Figure S3. Nucleotide diversity of mangroves in three regions and across species. Segments larger than 200 bp are shown. Upper and lower 'hinges' correspond to the first and third quartiles; the central line indicates the median. Asterisks represent diversity of species. Stars indicate highest π for *Ae. corniculatum*.

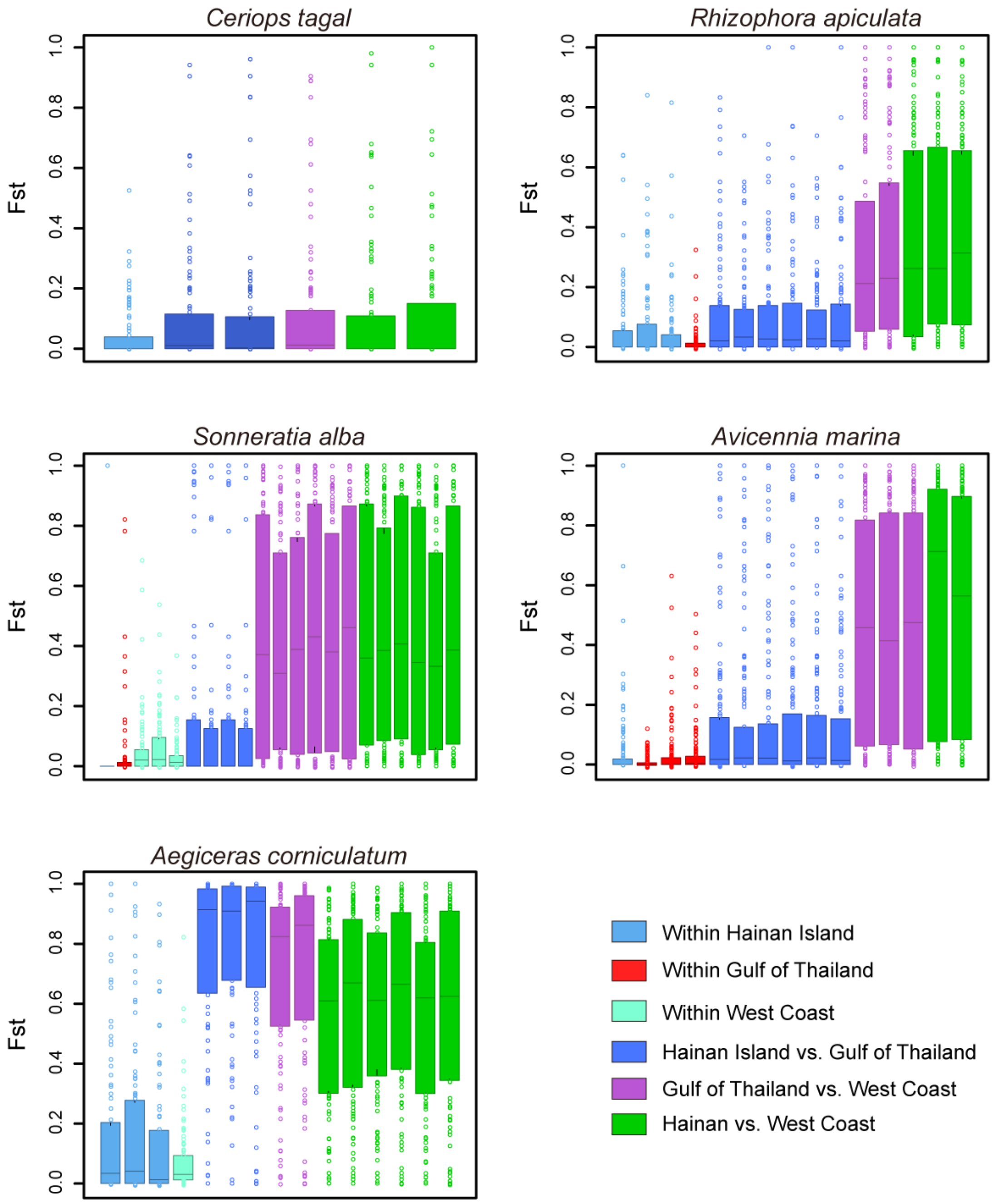

Figure S4. Genetic differentiation between mangrove populations. Genetic differentiation between populations is represented by F_{ST} for each locus. The columns on the left side of the vertical line show population differentiation within a region, the columns on the right show differentiation between regions.

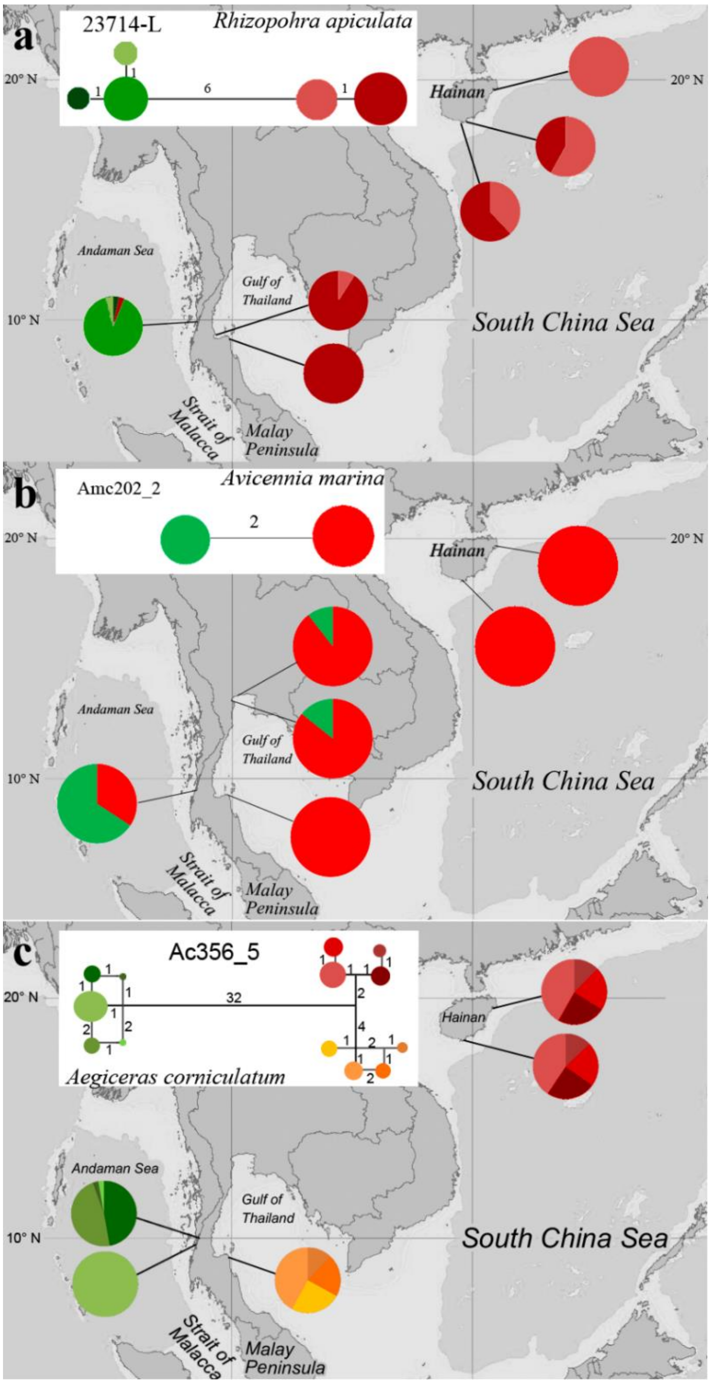

Figure S5. Three cases of haplotype structure. (a to **c)** the haplotype structures from *R. apiculata*, *Av. marina* and *Ae. corniculatum*.

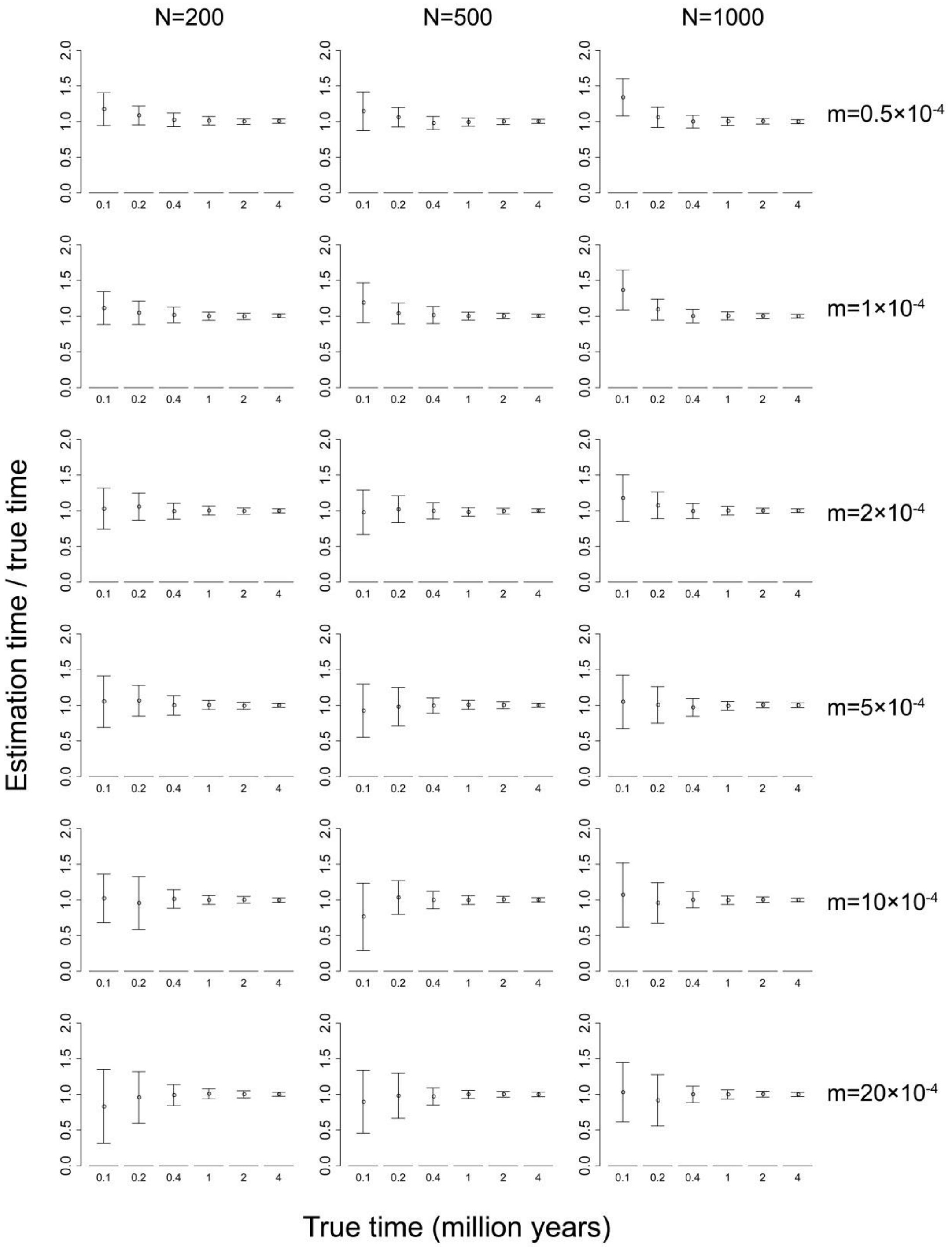

Figure S6. Accuracy of estimation of isolation time by likelihood methods under the SIM model. Parameters were set to 200 – 1,000 for population size (N), 0.5 – 20×10^{-4} for migration (m), 0.1 – 4 million years for isolation time. Each set of parameters was simulated 1,000 times. Each point in a panel shows mean ratio of estimated to true isolation time.

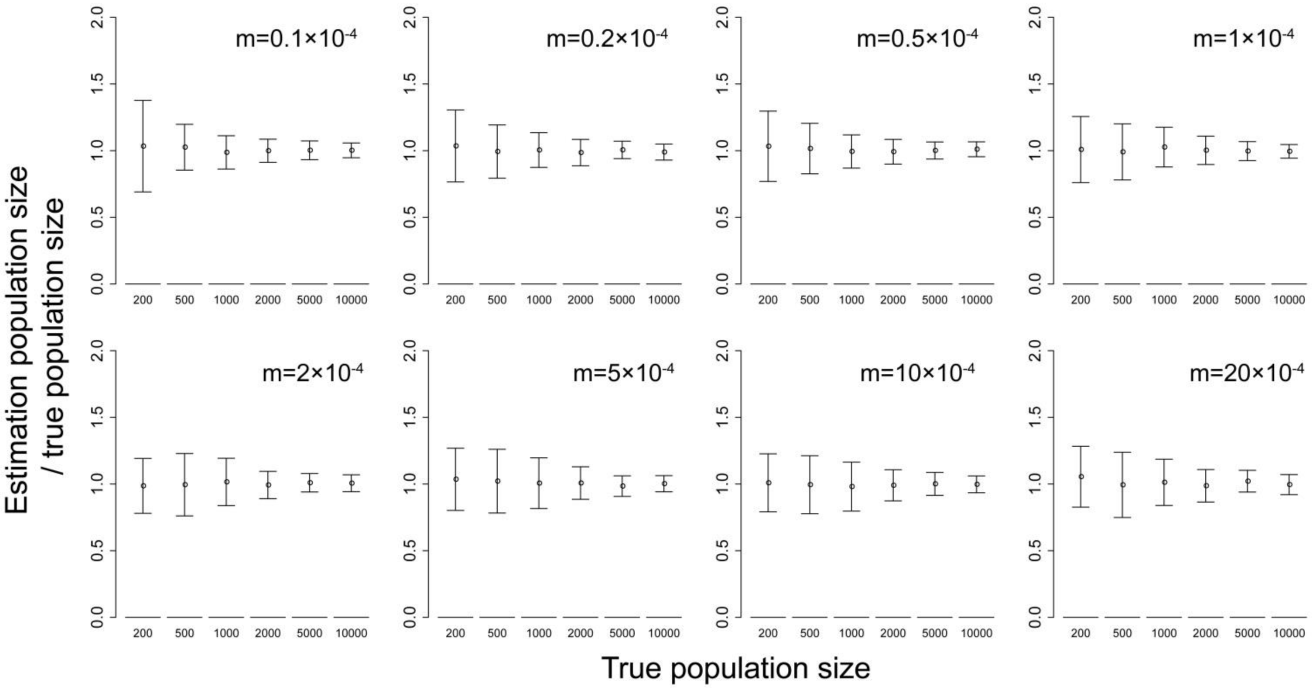

Figure S7. Accuracy of estimation of population size by likelihood methods under the MIM model. Parameters were set to 200 – 10,000 for population size (N) and 0.1 – 20×10^{-4} for migration (m). Each set of parameters was simulated 1,000 times. Each point in a panel shows mean ratio of estimated to true population size.

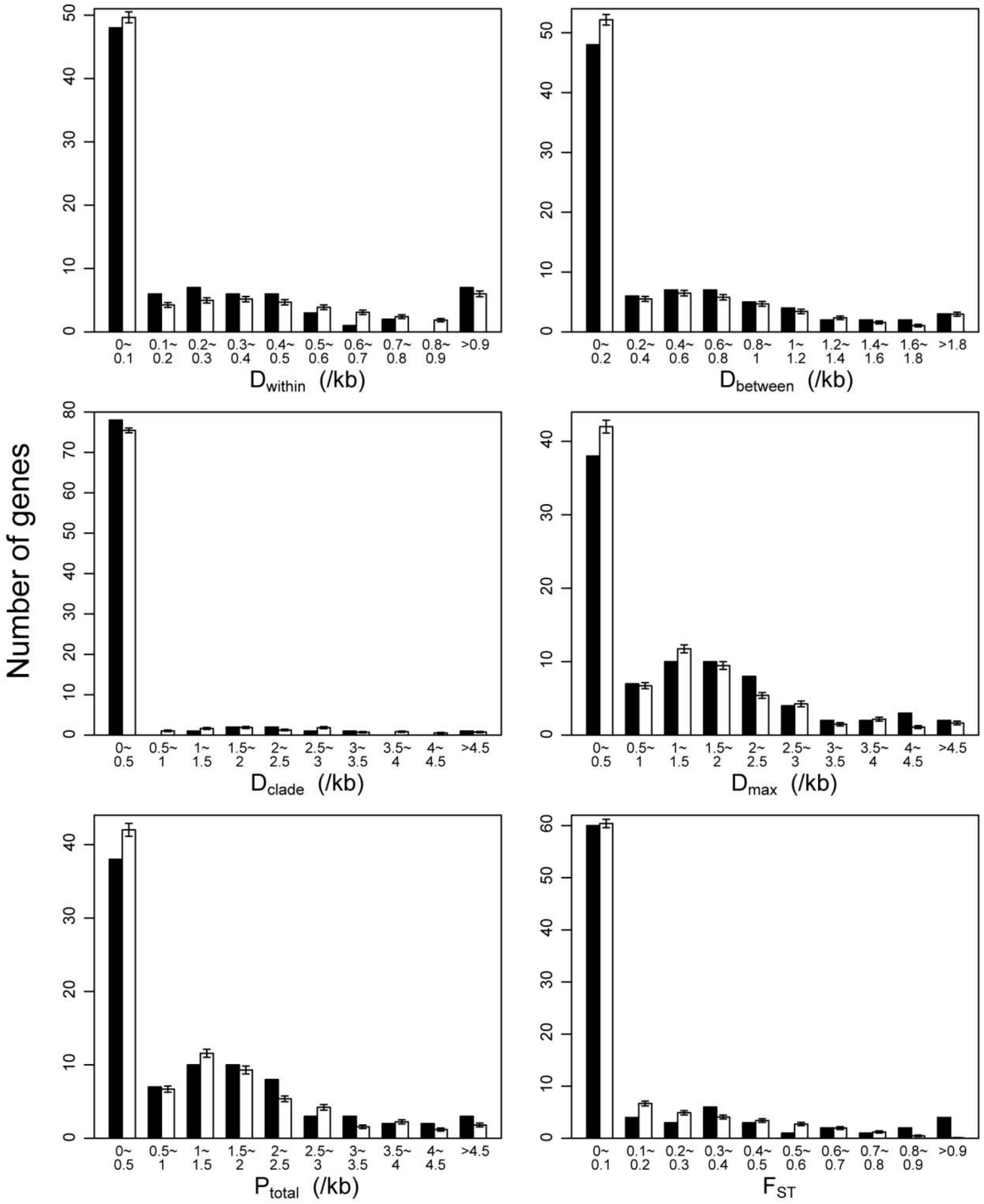

Figure S8. Comparison of six statistics from simulations under the MIM model to values observed in *C. tagal*. Solid bars show observations of six statistics: D_{within} (average differences within region), $D_{between}$ (average differences between regions), D_{clade} (differences between the most recent common ancestors of each clade), D_{max} (differences between two most divergent haplotypes), P_{total} (total number of SNPs), and F_{ST} between regions. Open bars show 2,000 replicate simulation results.

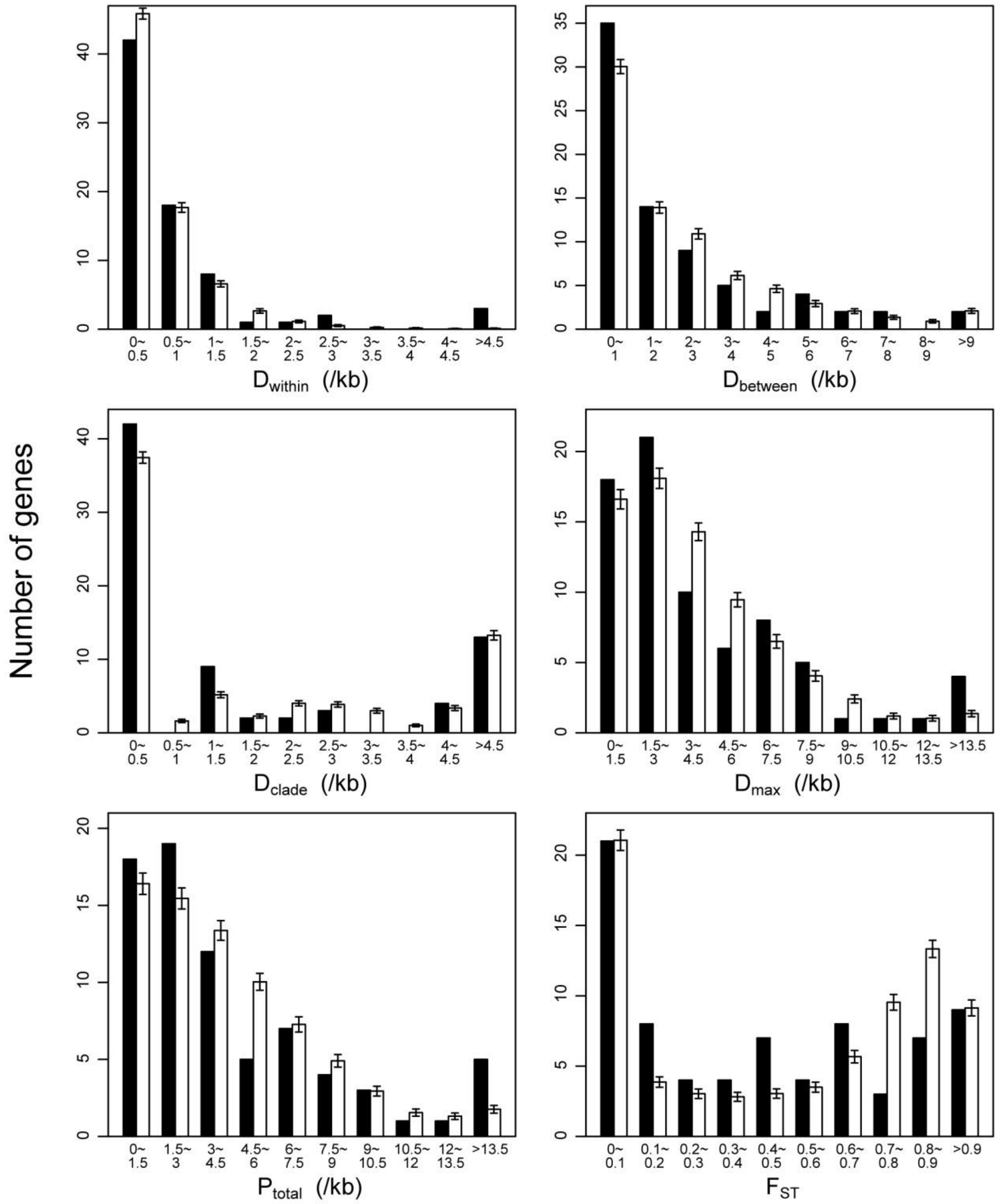

Figure S9. Comparison of six statistics from simulations under the MIM model to values observed in *R. apiculata.*

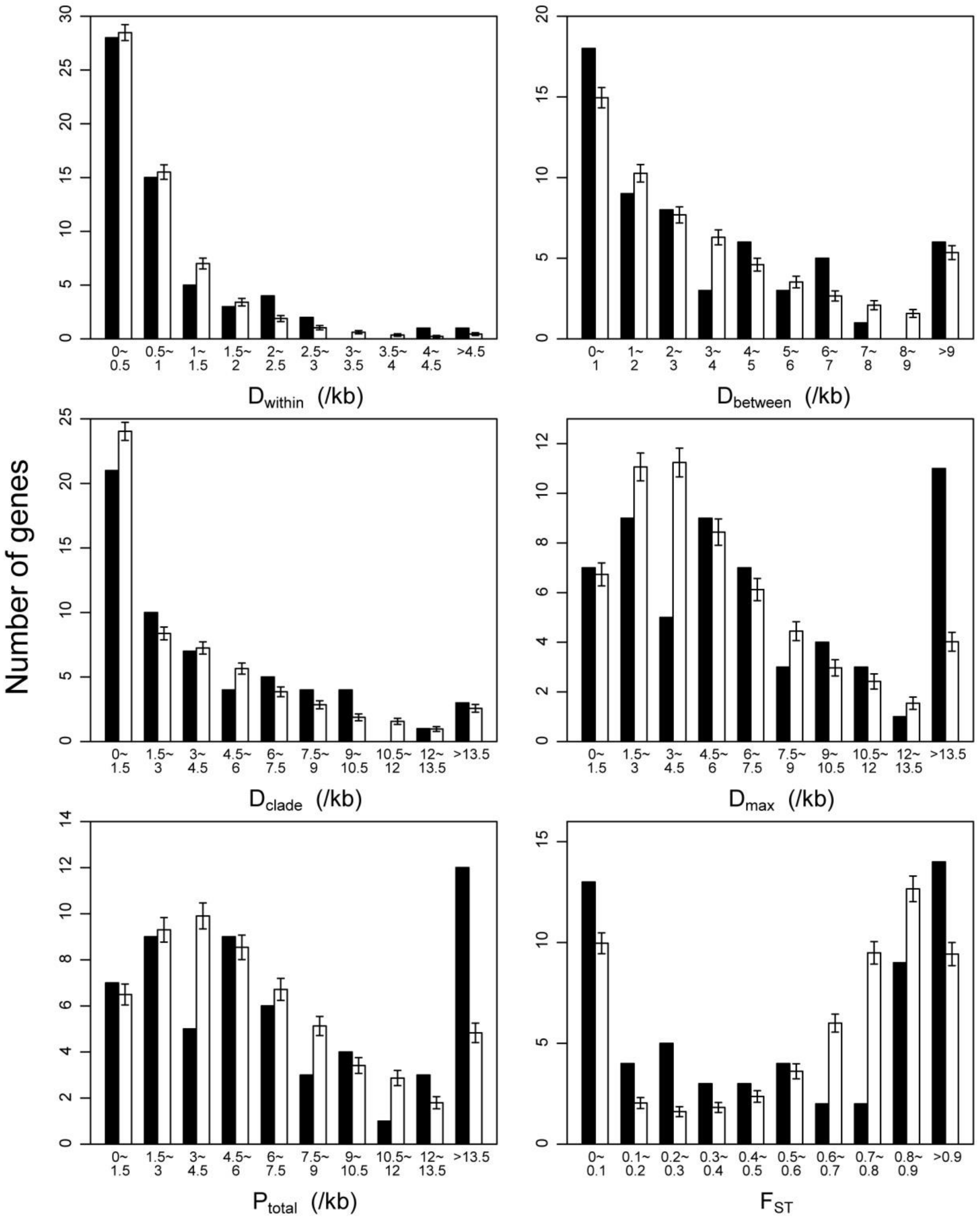

Figure S10. Comparison of six statistics from simulations under the MIM model to values observed in *S. alba.*

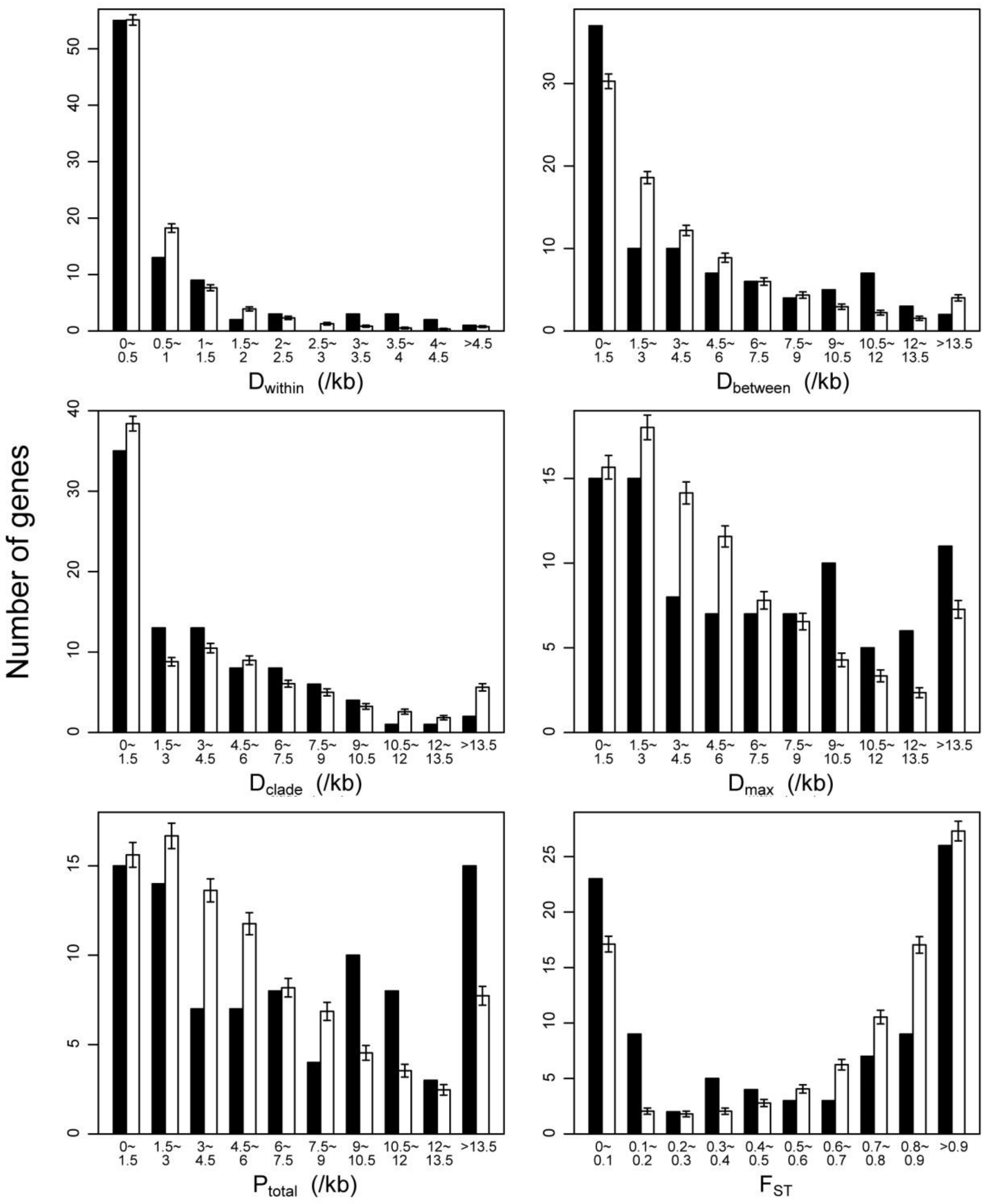

Figure S11. Comparison of six statistics from simulations under the MIM model to values observed in *Av. marina.*

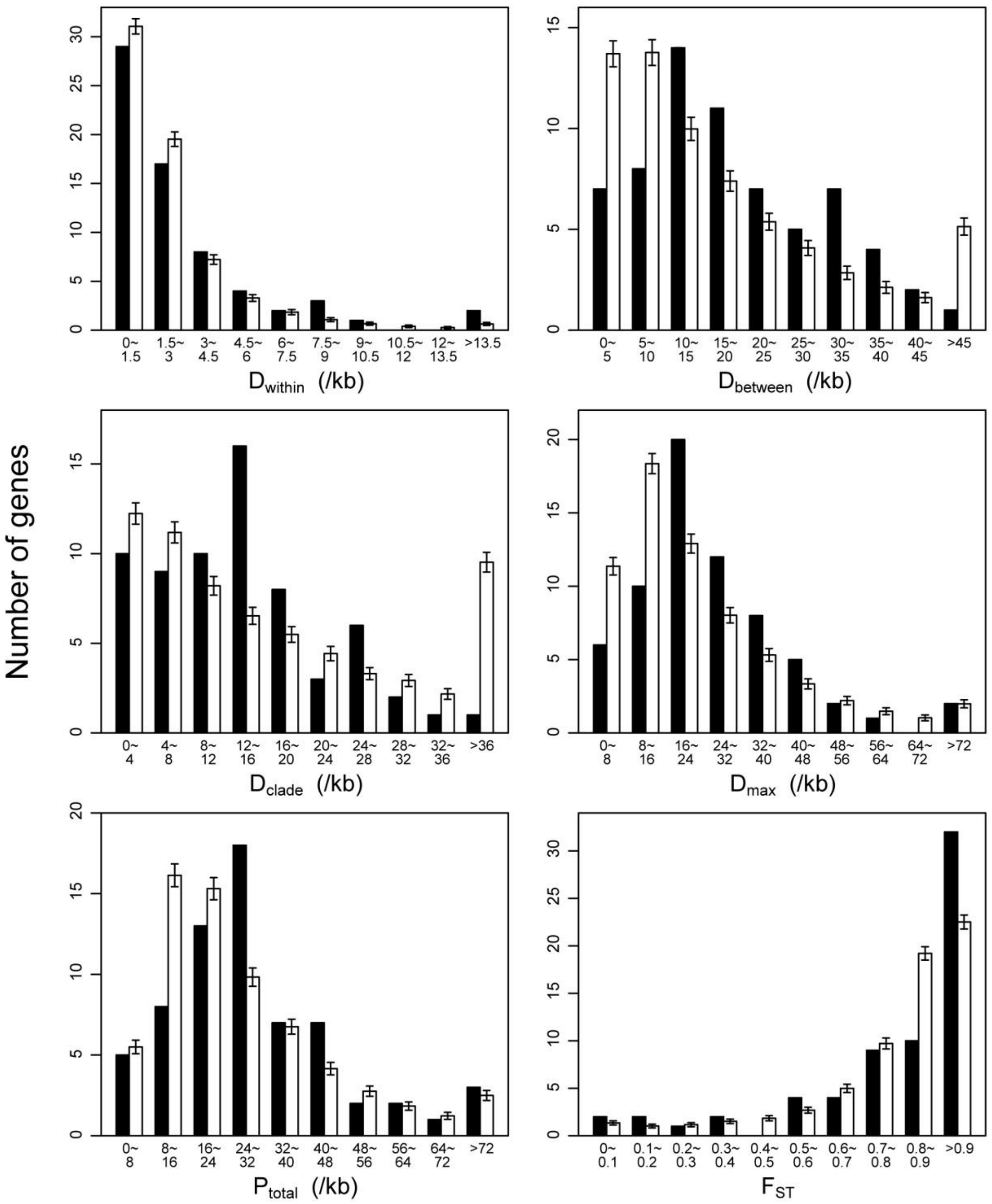

Figure S12. Comparison of six statistics from simulations under the MIM model to values observed in *Ae. corniculatum.*

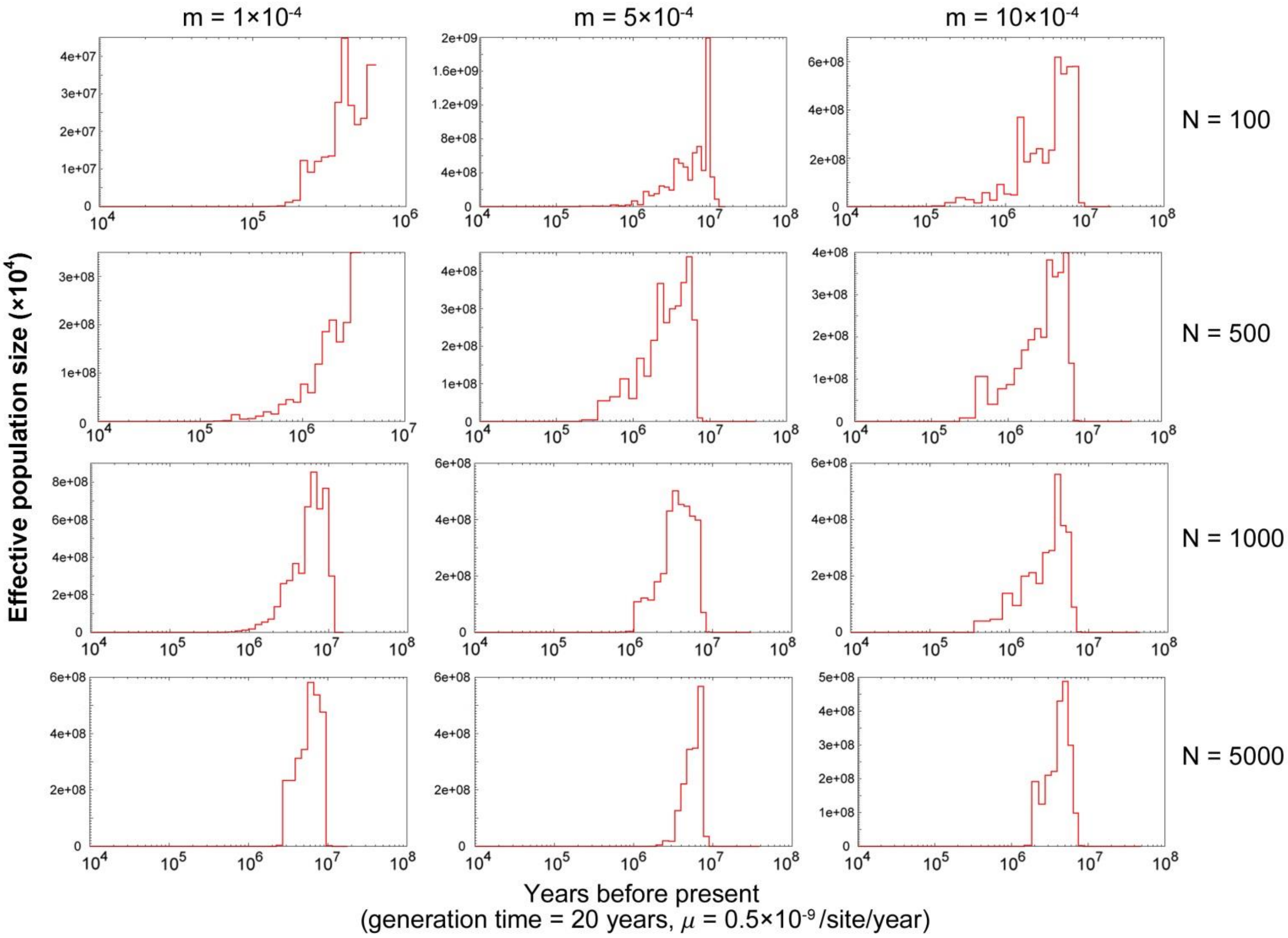

Figure S13. Estimation of effective population size changes under the SIM model. Mutation rate μ is set at 0.5×10^{-9} /site/year. Migration rate m is from 1×10^{-4} to 10×10^{-4} per generation and population size N is from 100 to 5,000.

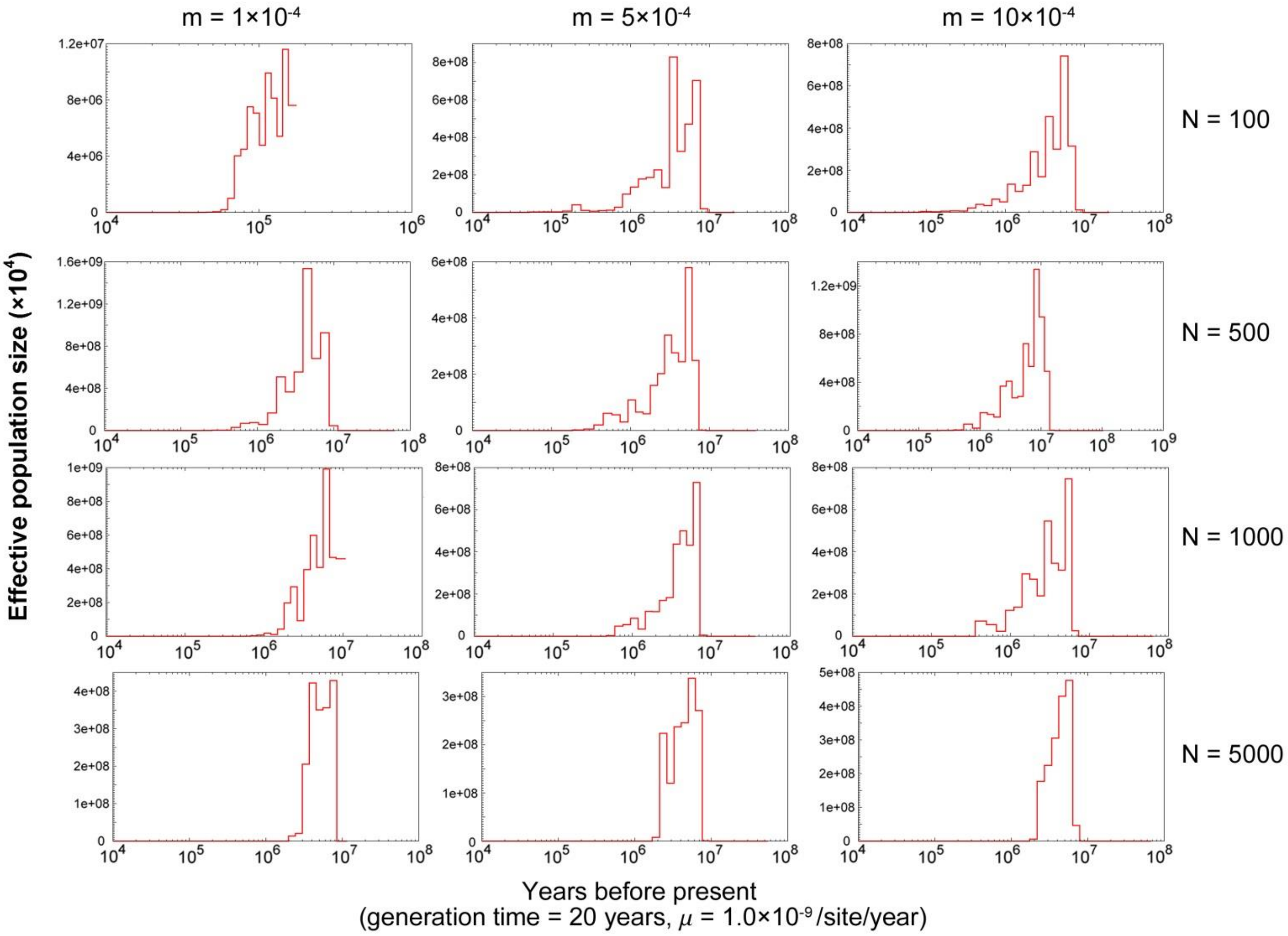

Figure S14. Estimation of effective population size change under the SIM model through simulation. Mutation rate μ is set at 1×10^{-9} /site/year. Migration rate m is from 1×10^{-4} to 10×10^{-4} per generation and population size N is from 100 to 5,000.

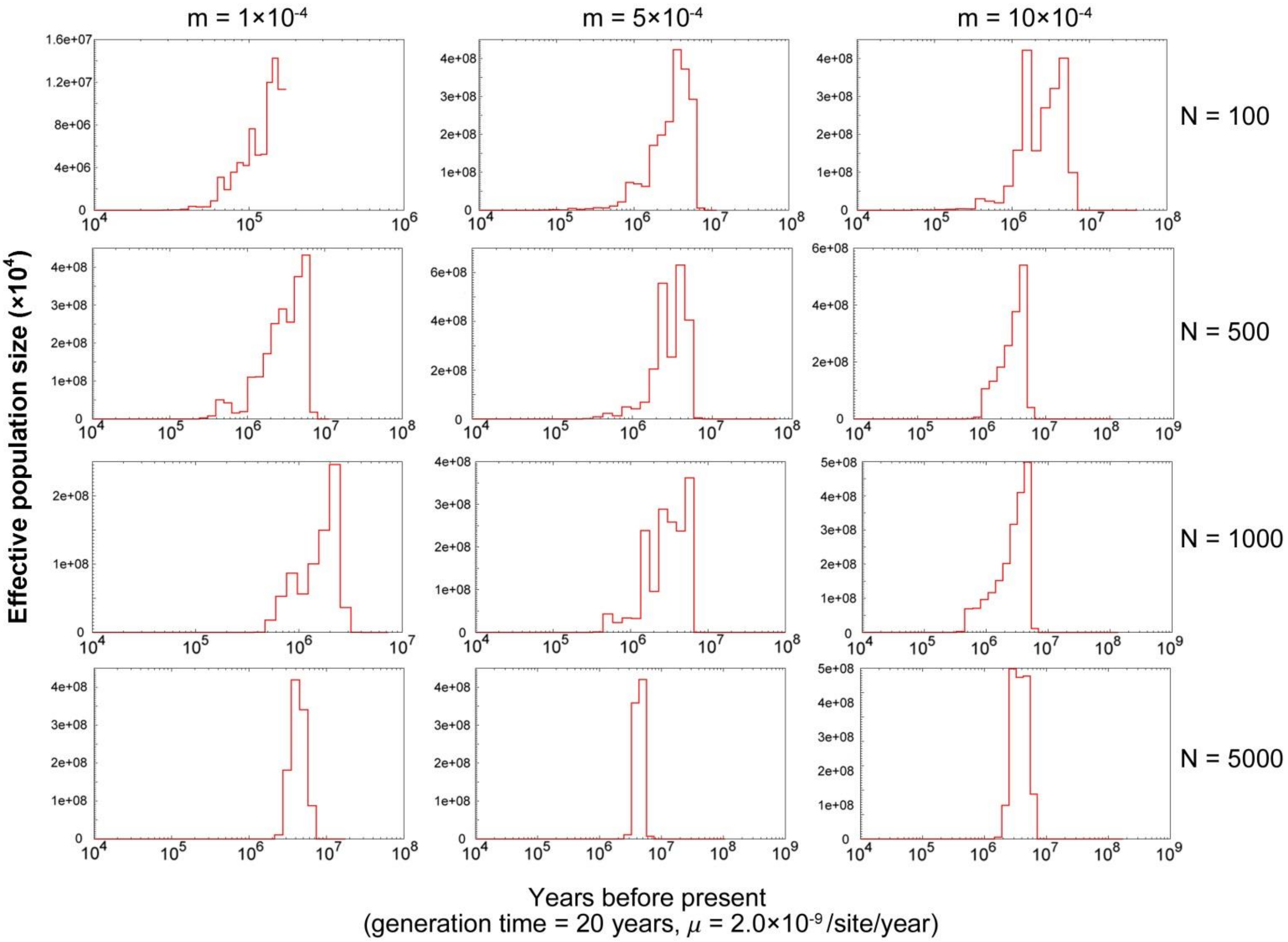

Figure S15. Estimation of effective population size change under the SIM model through simulation. Mutation rate μ is set at 2×10^{-9} /site/year. Migration rate m is from 1×10^{-4} to 10×10^{-4} per generation and population size N is from 100 to 5,000.

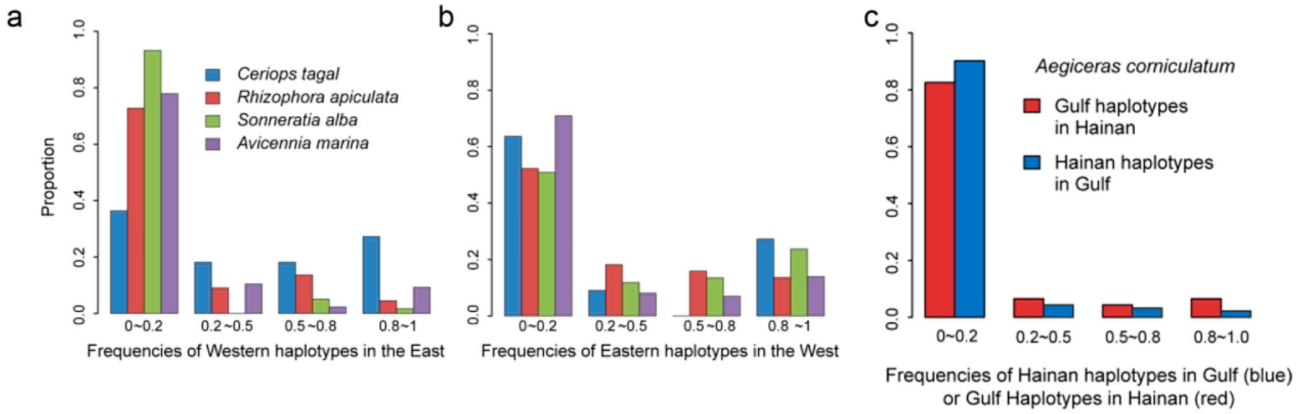

Figure S16. (a and **b**) Introgression of haplotypes between the East and West populations of four species. (**c**) In *Ae. corniculatum*, the genetic diversity is large and the partition can be seen even within the Eastern region. The Eastern haplotypes are divided into the Hainan type and Gulf type for the analysis of introgression (see text).

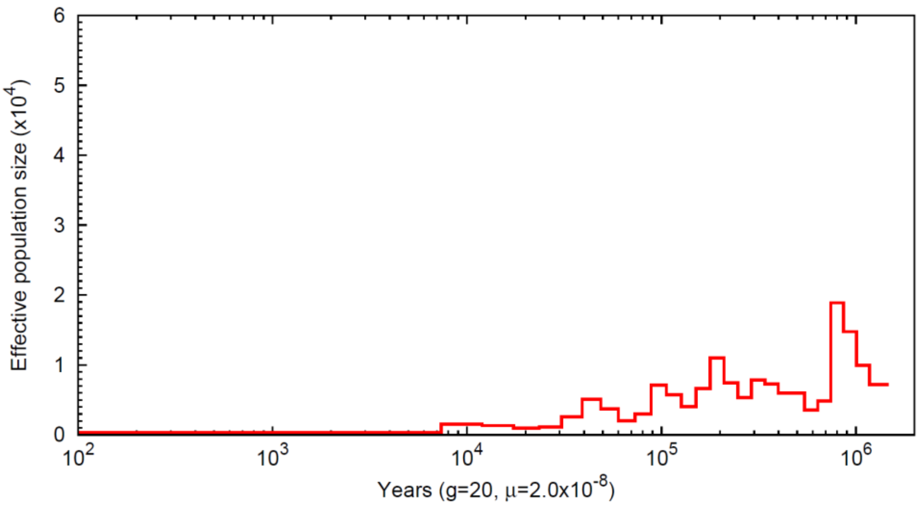

Figure S17. Estimation of effective population size change under the MIM model through simulation. Mutation rate μ is set at $1.0{\times}10^{-9}$ /site/year. The population size N is 1000 and migration Nm is 0.5, which is close the inferred values in Table 2.

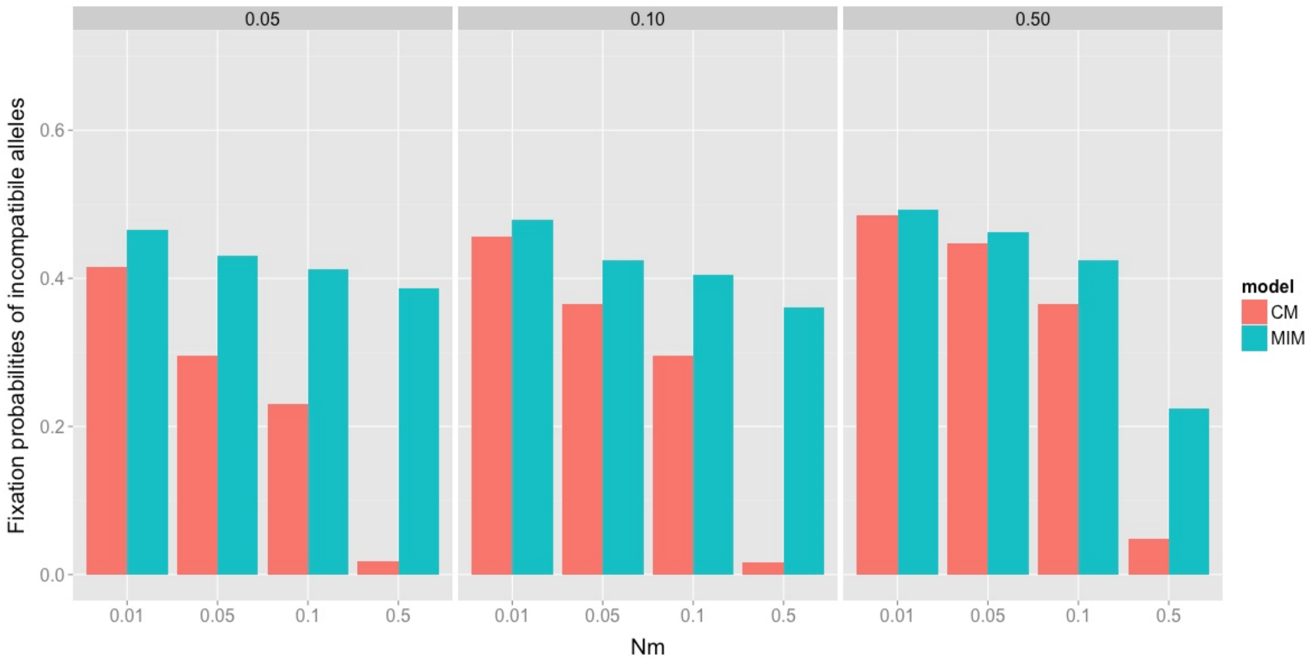

Figure S18. Simulation results for two-locus DMI. The fixation probabilities of two-locus DMI under CM model or MIM cycles model of a continent-island scenario for different levels of Nμ and Nm. Facet labels represent the different levels of Nμ values. The fixation probabilities are approximated by computer simulations. For two loci with two alleles each there are four genotypes under the haploid model: ab, Ab, aB and AB. The incompatibility interaction is between allele A and B. The fitness of ab, Ab and aB is equal to 1 but AB equal to 0. Mutation only happens from a to A or b to B at a rate of 1×10^{-3} /locus/generation and recombination rate is set to be 0. Under the continent-to-island unidirectional migration scenario, the continent is fixed for genotype aB and will not change during the evolutionary course. The island is composed of ancestral type ab initially and experiences evolutionary forces of selection, migration, recombination, mutation and genetic drift. Under the CM model migration is continuous while under the MIM cycles model the isolation phase lasts 900 generations and the migration phase lasts 100 generations per cycle. The average migration rate per cycle under the two models is the same. Genetic incompatibility is established when genotype Ab is fixed on the island. Thus, fixation probabilities are approximated by the number of simulations where Ab is fixed divided by the total number of simulations.

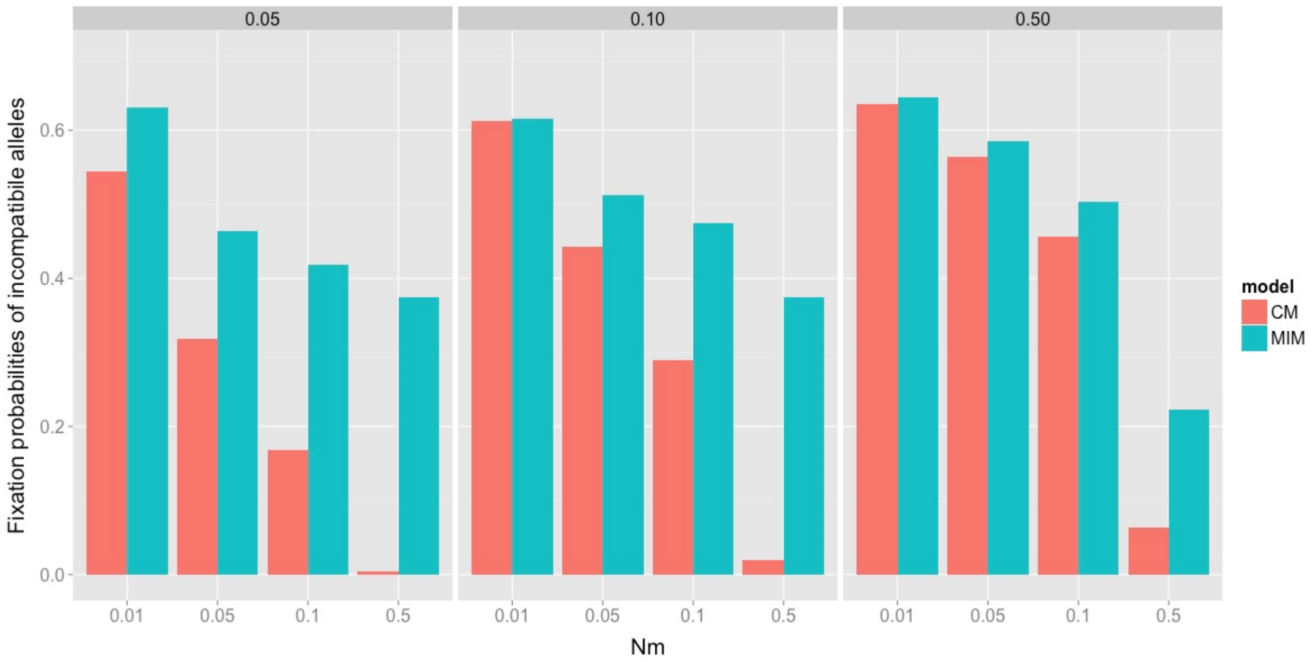

Figure S19. Simulation results for neutral three-locus DMI. The fixation probabilities of three-locus DMI under CM model or MIM cycles model of a continent-island scenario for different levels of Nμ and Nm. Facet labels represent the different levels of Nμ values. The fixation probabilities are approximated by computer simulations. For three loci with two alleles each there are eight genotypes under the haploid model: abc, Abc, aBc, abC, ABc, AbC, aBC and ABC. The incompatibility interaction is between AB and C. Thus, the fitness of ABC is 0 and all the other genotypes' fitness is equal to 1. Mutation only happens from a to A or b to B or c to C at a rate of 1×10^{-3} /locus/generation and recombination rate is set to be 0. Under the continent-to-island unidirectional migration scenario, the continent is fixed for genotype ABc and will not change during the evolutionary course. The island is composed of ancestral type abc initially and experiences evolutionary forces of selection, migration, recombination, mutation and genetic drift. Migration is continuous under the CM model, while the isolation phase lasts 900 generations and the migration phase lasts 100 generations per cycle under the MIM model. The average migration rate per cycle under the two models is the same. Genetic incompatibility is established when the genotype AbC or aBC is fixed on the island. Thus, fixation probabilities are approximated by the number of simulations where AbC or aBC is fixed divided by the total number of simulations.

References

93. Li L, Stoeckert CJ, & Roos DS (2003) OrthoMCL: Identification of ortholog groups for eukaryotic genomes. *Genome Res* 13(9):2178-2189.

94. Zhang Z*, et al.* (2006) KaKs_Calculator: Calculating Ka and Ks Through Model Selection and Model Averaging. *Genomics Proteomics Bioinformatics* 4(4):259-263.

95. Flaxman SM, Wacholder AC, Feder JL, & Nosil P (2014) Theoretical models of the influence of genomic architecture on the dynamics of speciation. *Mol Ecol* 23(16):4074-4088.

96. Nosil P & Feder JL (2012) Genomic divergence during speciation: causes and consequences. *Philos Trans Royal Soc B* 367(1587):332-342.

97. Via S (2012) Divergence hitchhiking and the spread of genomic isolation during ecological speciation-with-gene-flow. *Philos Trans Royal Soc B* 367(1587):451-460.

98. Clarke PJ, Kerrigan RA, & Westphal CJ (2001) Dispersal potential and early growth in 14 tropical mangroves: do early life history traits correlate with patterns of adult distribution? *J Ecol* 89(4):648-659.

99. Agren J & Schemske DW (2012) Reciprocal transplants demonstrate strong adaptive differentiation of the model organism Arabidopsis thaliana in its native range. *New Phytol* 194(4):1112-1122.

100. Clarke PJ (1995) The population dynamics of the mangrove shrub Aegiceras corniculatum (Myrsinaceae): fecundity, dispersal, establishment and population structure. *Proc Linn Soc NSW* 115:35-44.

101. Carneiro M*, et al.* (2014) The Genomic architecture of population divergence between subspecies of the European rabbit. *PLoS Genet* 10(8):e1003519.

102. Ellegren H*, et al.* (2012) The genomic landscape of species divergence in Ficedula flycatchers. *Nature* 491(7426):756-760.

103. Harr B (2006) Genomic islands of differentiation between house mouse subspecies. *Genome Res* 16(6):730-737.

104. Renaut S*, et al.* (2013) Genomic islands of divergence are not affected by geography of speciation in sunflowers. *Nat Commun* 4:1827.

105. Turner TL, Hahn MW, & Nuzhdin SV (2005) Genomic islands of speciation in Anopheles gambiae. *PLoS Biol* 3(9):1572-1578.

106. Ting C-T, Takahashi A, & Wu C-I (2001) Incipient speciation by sexual isolation in Drosophila: concurrent evolution at multiple loci. *Proc Natl Acad Sci USA* 98(12):6709-6713.

107. Rundell RJ & Price TD (2009) Adaptive radiation, nonadaptive radiation, ecological speciation and nonecological speciation. *Trends Ecol Evol* 24(7):394-399.

108. Kimura M & King JL (1979) Fixation of a deleterious allele at one of two" duplicate" loci by mutation pressure and random drift. *Proc Natl Acad Sci USA* 76(6):2858-2861.

109. Li W-H (1980) Rate of gene silencing at duplicate loci: a theoretical study and interpretation of data from tetraploid fishes. *Genetics* 95(1):237-258.

110. Takahata N & Maruyama T (1979) Polymorphism and loss of duplicate gene expression: a theoretical study with application of tetraploid fish. *Proc Natl Acad Sci USA* 76(9):4521-4525.

111. Wu C-I & Palopoli MF (1994) Genetics of postmating reproductive isolation in animals. *Annu Rev Genet* 28(1):283-308.

112. Zhou R, *et al.* (2007) Population genetics of speciation in nonmodel organisms: I. Ancestral polymorphism in mangroves. *Mol Biol Evol* 24(12):2746-2754.

113. Yang YC, *et al.* (2015) Phylogenetic position of Sonneratia griffithii based on sequences of the nuclear ribosomal internal transcribed spacer and 13 nuclear genes. *J Syst Evol* 53(1):47-52.

114. Urashi C, Teshima KM, Minobe S, Koizumi O, & Inomata N (2013) Inferences of evolutionary history of a widely distributed mangrove species, Bruguiera gymnorrhiza, in the Indo-West Pacific region. *Ecol Evol* 3(7):2251-2261.

115. Inomata N, Wang X-R, Changtragoon S, & Szmidt AE (2009) Levels and patterns of DNA variation in two sympatric mangrove species, Rhizophora apiculata and R. mucronata from Thailand. *Genes Genet Syst* 84(4):277-286.

116. Ng WL & Szmidt AE (2015) Introgressive hybridization in two Indo-West Pacific Rhizophora mangrove species, R. mucronata and R. stylosa. *Aquat Bot* 120:222-228.

117. Huang Y, *et al.* (2012) Differentiated population structure of a genetically depauperate mangrove species Ceriops tagal revealed by both Sanger and deep sequencing. *Aquat Bot* 101:46-54.
